# Citrate-EDTA-H_2_O_2_ buffering leaching solution for Ni/Co/Mn recovery from spent lithium-ion battery black mass

**DOI:** 10.1039/d5ra06978e

**Published:** 2025-10-27

**Authors:** Saken Abdimomyn, Zhulduz Zhanatkyzy, Grigoryev Artur, Seilbek Malik, Kayirgali Zhumadil, Sergey Nechipurenko, Fyodor Malchik

**Affiliations:** a Faculty of Chemistry and Chemical Technology, Al-Farabi Kazakh National University Al-Farabi 71/23 Almaty Kazakhstan frodo-007@mail.ru abdimomyn03@gmail.com zhuldyzjjj@mail.ru artur.grigoryev8@gmail.com seilbekmalik@gmail.com maldybayevkaiyrgali@gmail.com nechipurenkos@mail.ru

## Abstract

The increasing global demand for lithium-ion batteries necessitates the development of environmentally sustainable recycling technologies for critical metal recovery. This study presents a novel citrate-EDTA buffered leaching system for recovery of Li, Co, Ni, and Mn from spent LIB cathode materials with mixed NMC/LiCoO_2_ composition. The developed approach addresses limitations of conventional citric acid leaching through synergistic combination of citrate buffer (pH 4–6), Na_2_EDTA as complexing agent, and H_2_O_2_ as reducing agent under mild conditions (50 °C). Thermodynamic analysis using Pourbaix diagrams demonstrated that the citrate-EDTA system significantly enhances metal solubility by forming stable chelate complexes and shifting redox boundaries to prevent passivation layer formation. Key parameters were optimized using response surface methodology and central composite rotatable plan to maximize metal recovery: 1.211 v/v% H_2_O_2_, 0.778 mol L^−1^ citrate buffer, and 0.05 mol L^−1^ Na_2_EDTA. Kinetic studies revealed maximum metal leaching efficiencies at pH 5.0, solid-to-liquid ratio 1 : 20, and temperature 50 °C of Li—100.0%, Co—98.65%, Ni—90.69%, and Mn—82.87% under these mild conditions. Kinetic modeling using Avrami–Erofeev and Peleg equations revealed distinct leaching mechanisms: rapid delithiation followed by interfacial reaction control for Li and Co, while Ni and Mn exhibited diffusion-limited behavior with passivation effects. Comparative analysis demonstrated that the developed system is nearly as effective as traditional acid methods when operating at lower temperatures with less impact on the environment. Thermodynamic barrier analysis revealed the activation energy sequence: Co (92.1) > Ni (87.4) ≈ Mn (87.2) > Li (83.25) kJ mol^−1^, confirming the mechanistic insights. This green chemistry approach offers significant advantages, including biocompatibility, mild operating conditions, and potential for industrial scale-up in sustainable battery recycling applications.

## Abstract 2

Citrate-EDTA-H_2_O_2_ buffering system recovers Li/Co/Ni/Mn from battery black mass at pH 5.0 using a dual chelation mechanism.

## Graphical abstract

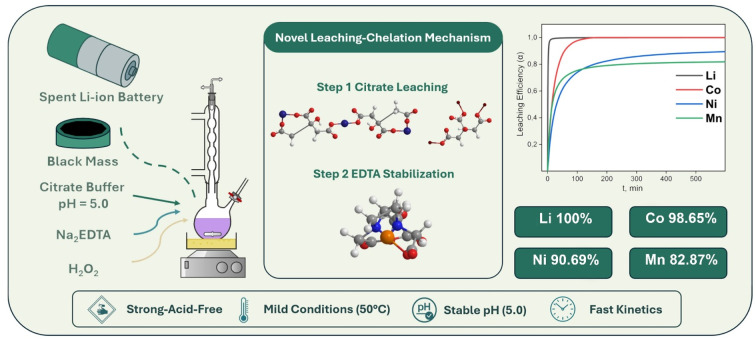

## 1 Introduction

The growing demand for environmentally sustainable energy solutions has driven the intensive development of the lithium-ion battery (LIB) recycling industry, which has become a critically important element of global innovation and economic transformations.^1^ The expansion of electric vehicles, renewable energy systems, and consumer electronics has led to LIB recycling being recognised as a key solution to resource scarcity problems and environmental challenges.^2^ The recycling market is defined by tightening environmental regulation, the need for decarbonisation of electric vehicle supply chains, increasing volumes of end-of-life batteries, and rising demand for critical materials – Li, Co, Ni, and Mn.^3^

**Fig. 1:**
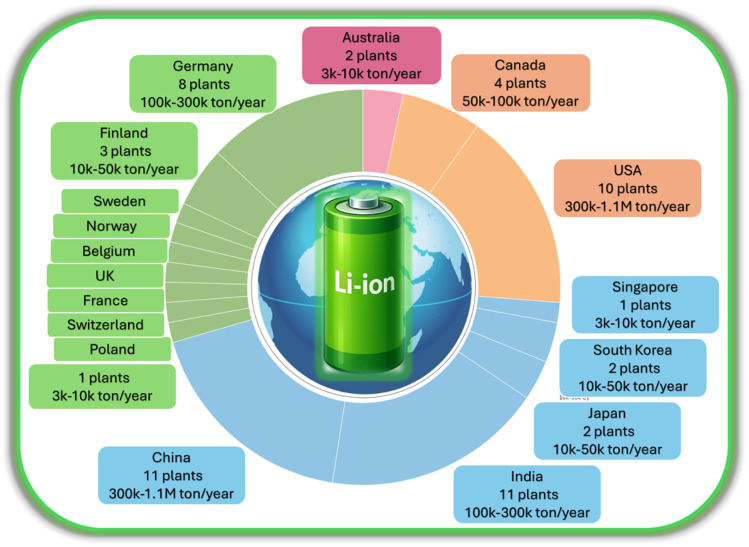
Geographical distribution of established recycling facilities for LIBs.^8^

In accordance with sustainable development concepts, over the past three years, the governments of China, the USA, the EU, South Korea, and India have implemented stricter regulatory standards for waste recycling systems aimed at reducing the carbon footprint of industrial enterprises.^4–7^ These measures have resulted in rapid development of the global LIB recycling industry (Fig. 1). The aggregate capacity of operational facilities exceeds 1.6 million tonnes per year, and with construction projects underway, growth to 3 million tonnes per year is anticipated.^8,9^

Among the three main approaches for lithium-ion battery recycling pyrometallurgy,^10,11^ hydrometallurgy^2,12^ and direct recycling^13,14^ – hydrometallurgy stands out as the most promising solution in terms of environmental impact, economic efficiency, and technological flexibility. The leaching process constitutes the primary stage in hydrometallurgical LIB recycling.^15^ Traditionally, inorganic/mineral acids such as H_2_SO_4_,^16–20^ HCl,^21–23^ HNO_3_,^24^ H_3_PO_4_ (ref. 25 and 26) and HF^27^ are employed for metal recovery from spent LIBs black mass.

In connection with the transition to energy-efficient technologies, organic acids have gained popularity in spent lithium-ion battery recycling. Their advantages include: environmental safety (lower toxicity, biodegradability), mild conditions (pH 1–5), and economic benefits. To enhance leaching rates, reducing agents such as H_2_O_2_,^28^ NaHSO_3_,^29^ Na_2_S_2_O_5_,^30^ NH_2_OH^31^ и Na_2_SO_3_,^32^ are added, facilitating the conversion of Co^3+^, Ni^3+^, and Mn^4+^ to soluble divalent ions. Unlike inorganic acids, which leach all metals with low selectivity,^33^ organic acids can function as solvent, reducing agent, precipitant, or complexing agent.

Citric acid (C_6_H_8_O_7_) is a widely applied organic reagent in hydrometallurgical black mass recycling due to its availability, biodegradability, complexing ability, and environmental safety. Due to its three-stage dissociation, citric acid can be utilised across a broad range of solution pH values (Table 1).^34,35^

**Table 1:** Dissociation constants of citric acid^36^

Dissociation equation	Dissociation constants	
	*K*_a_	p*K*_a_
H_3_Cit ⇌ H_2_Cit^−^ + H^+^	7.4 × 10^−4^ (*K*_1_)	3.13
H_2_Cit^−^ ⇌ HCit^2−^ + H^+^	1.7 × 10^−5^ (*K*_2_)	4.76
HCit^2−^ ⇌ Cit^3−^ + H^+^	4.0 × 10^−7^ (*K*_3_)	6.40

Citric acid is a weak organic acid with limited water solubility, which may constrain its leaching properties. The interaction of citric acid with LiCoO_2_ results in the formation of trivalent cobalt (Co^3+^), characterized by low solubility in aqueous solutions and reduced leaching process efficiency. This problem is resolved through the application of reducing agents, ensuring the reduction of Me^4+/3+^ to Me^2+^, which significantly enhances metal solubility and leaching efficiency.^33^

The prevailing trend in literature data dictates that high concentrations of citric acid (0.5–4.0 mol L^−1^) and acidic conditions (pH = 1.0–2.0) are employed, where leaching efficiency depends primarily on acidic nature (p*K*_a_) and unique chelating coordination properties (p*K*_β_) (Table 2). The use of high temperatures (50–90 °C) and H_2_O_2_ concentrations (1–10 vol%) results in reagent costs and, consequently, a lack of technological viability. Furthermore, instability of Me^2+^ ions in weakly acidic and neutral media, due to exposure to atmospheric CO_2_ and O_2_ and hydrolysis, may lead to losses during subsequent concentration stages:

1 2Co_3_(CitH)_2_ +3O_2_ +2H_2_O → 6Co(OH)_3_↓ + 4H_3_Cit (pH ≥ 6)

2 2Co_3_(CitH)_2_ + 3O_2_ + 6H^+^ → 6Co^3+^ + 4H_3_Cit (pH < 6)

3 6Co^3+^ + 9H_2_O → 6Co(OH)_3_↓ + 9H ^+^

4 6Co(OH)_3_ → Co_2_O_3_ + H_2_O

**Table 2:** Summary of conditions and leaching efficiencies of citric acid used for the leaching of spent LIB black masses

Cathode material	Leaching agent (conditions)	Leaching efficiency	Ref.
LiCoO_2_	Citric acid 4 mol L^−1^ + H_2_O_2_ 1 vol%; S/L 15 g L^−1^; 90 °C; 5 h	Co 99.07%	37
LiCoO_2_	Citric acid 1.25 mol L^−1^ + H_2_O_2_ 1 vol%; S/L 20 g L^−1^; 90 °C; 30 min	Co 90%, Li 100%	38
LiCoO_2_	Citric acid 1 mol L^−1^ + H_2_O_2_ 8 vol%; S/L 40 g L^−1^; 70 °C; 70 min	Co 99%, Li 99%	39
LiCoO_2_	Citric acid 1.25 mol L^−1^ + H_2_O_2_ 0.9 vol%; S/L 60 g L^−1^; 90 °C; 35 min	Co 90.2%, Li 98%	40
LiCoO_2_	Citric acid 2.0 mol L^−1^ + H_2_O_2_ (reductant dose 0.6 g g^−1^); S/L 50 g L^−1^; 70 °C; 80 min	Co ∼98%, Li ∼99%	41
LiCoO_2_	Citric acid 1.5 mol L^−1^ + tea waste 0.4 g g^−1^; S/L 30 g L^−1^; 90 °C; 120 min	Co 96%, Li 98%	41
LiCoO_2_	Citric acid 1.5 mol L^−1^ + PA 0.4 g g; S/L 40 g L; 80 °C; 120 min	Co 83%, Li 96%	41
Mixed cathode active materials (industrial waste)	Citric acid 0.5 mol L^−1^; S/L 80 g L^−1^; 90 °C; 80 min; no reductant	Li 91.0%, Co 90.9%, Ni 94.1%, Mn 88.6%, Cu 19.5%, Al 26.9%	42
Mixed cathode active materials	Citric acid 1.5 mol L^−1^ + H_2_O_2_ 2 vol%; 95 °C; 30 min	Co 98%, Li 96%, Ni 99%	43
Various cathode active materials	Citric acid 2 mol L^−1^ + H_2_O_2_ 0.25 M (composition evaluation)	Co 106%, Ni 90%, Mn 92%, Cu 94%, Al 93%	44
Mixed electrode masses	Citric acid/H_2_O_2_ system (selective for Co and Ni)	Co 83%, Ni 100%, Mn 30%, Cu 78%, Al 3%	45
Commercial collected cathode active materials	Citric acid 1 mol L^−1^ + H_2_O_2_ 1 vol%; S/L 50 g L^−1^; ∼25 °C; 24 h	Co 97%, Li 89%, Mn 98%, Ni 93%	46
S-LIB cathode active materials (mixed)	Citric acid 1.5 mol L^−1^ + d-glucose 0.5 g g^−1^; S/L 20 g L; 80 °C; 2 h	Li 99%, Ni 91%, Co 92%, Mn 94%	47

In response to the disadvantages of traditional citric acid leaching, the novelty of this work lies in the application of a system based on a low-concentration citrate buffer (pH 4.0–6.0) with the addition of Na_2_EDTA as a complexing agent and H_2_O_2_ as a reducing agent. For the first time, the use of citrate buffer as a source of citrate anions combined with EDTA^2−^ as a stabilizing agent to prevent Me^2+^ hydrate formation is proposed. This approach will enable enhancement of metal leaching efficiency above 90% and ensure stabilization of complex ions in the leaching solution.

## 2 Experimental part

### 2.1 Preparation of the studied electrode mass material

The end-of-life 16 350-type LIBs used in this study were sourced from disposable consumer electronics, specifically portable vaporisers of one type. Pre-cleaned and discharged using a CT-4008-5V10A battery tester, the batteries were subjected to grinding using a laboratory-type shredder. To remove residual organic electrolyte, the ground mixture was dried in a drying oven at 50 °C for 2.5 hours. The dried powder was subjected to fractionation on a CISA PR-200N laboratory vibrating sieve. The electrode mass fraction <100 μm was subjected to annealing at 600 °C for 15 hours in a muffle furnace to remove organic electrolyte and membrane components. The <100 μm fraction after annealing was used for the leaching process (Fig. S1).

### 2.2 Determination of phase and elemental composition of the annealed electrode mass

The phase composition study was performed using X-ray diffraction analysis on a Tongda TD-3700 X-ray diffractometer (China), equipped with a copper anode X-ray tube (CuKα radiation, *λ* = 1.5418 Å).

The mass fraction of components was determined by “wet chemistry”. A 2.5 g sample was leached at 70 °C in 50.0 ml of 2 mol L^−1^ H_2_SO_4_ with 5 v/v% H_2_O_2_ (S : L = 1 : 20, 15 h): after brief stirring (5–7 s), the suspension was kept in an ultrasonic bath for 1 h (70 °C), then stirred on a magnetic stirrer (400 rpm, 15 h). Upon completion, vacuum filtration was performed through PVDF membranes; the residue was repeatedly washed with water and dried for 1 h in a vacuum oven. The filtrate was analyzed by atomic adsorbtion spectroscopy (AAS) (Shimadzu AA-6200, Japan) for main metals (Li, Co, Ni, Mn, Fe and Cu). Experiments for elemental composition determination were conducted in triplicate.

### 2.3 Leaching in citrate buffer system with addition of H_2_O_2_ and Na_2_EDTA

A citrate buffer based on citric acid monohydrate (C_6_H_8_O_7_·H_2_O) and trisodium citrate (Na_3_C_6_H_5_O_7_·2H_2_O) was used as the leaching agent; H_2_O_2_ was used as a reducing agent; disodium salt of ethylenediaminetetraacetic acid (Na_2_EDTA) was used as a complexing agent. All other reagents used were of analytical grade, and all solutions were prepared or diluted with distilled water.

All leaching experiments were conducted in a three-neck round-bottom flask (150 ml capacity) with a reflux condenser to prevent losses due to evaporation. In 25.0 ml of citrate buffer solution with additives (H_2_O_2_, Na_2_EDTA) at a fixed temperature, 0.5000 g of homogenized electrode mass was loaded, and the suspension was stirred at 400 rpm. A water bath was used to control the reaction temperature. To obtain a representative sample and ensure uniform distribution of components in the electrode mass, quartering was performed using the “ring-cone” method for each studied sample.

### 2.4 Experimental design and response surface methodology

Response Surface Methodology (RSM) was used to optimize reagent regime of the Li, Co, Ni and Mn leaching and to evaluate the effect of factors on process efficiency. The experimental design was built based on a Central Composite Rotatable Design (CCRD).^48^ All experiments were conducted with constant temperature = 50 °C, S : L = 1 : 20 and initial pH = 5.0. This approach reveals nonlinear effects of factors on leaching efficiency and maintains uniform prediction variance at equal distances from the center of the design (rotatability). The study included 3 controllable factors: *A* – volume fraction of hydrogen peroxide (H_2_O_2_, v/v, %), *B* – total molar concentration of citrates (citric acid/sodium citrate buffer solution, M), *C* – molar concentration of Na_2_EDTA (M). The design consists of five coding levels (−*α*, −1, 0, +1, +*α*), where *α* = 1.682. The total number of experiments was calculated using the formula (5):

5 *N* = 2^*k*^ + 2*k* + *n*_c_

where *N* – total number of experiments, *k* – number of factors and *n*_c_ – number of replications at the central point. The total number of experiments was 19, including 8 cube vertices (factorial points), 6 axial (“star”) points and 5 center point replications to estimate pure error.^49^ The factor levels in natural and coded form are presented in Table 3.

**Table 3:** Independent variables and factor levels

Factor	Name	Units	Uncoded levels				
			−*α*	−1	0	+1	+*α*
*A*	Concentration of H_2_O_2_	v/v%	0.05	1.05	2.53	4.00	5.00
*B*	Concentration of citric anion	M	0.05	0.24	0.53	0.81	1.00
*C*	Concentration of Na_2_EDTA	M	0.01	0.05	0.11	0.16	0.20

The leaching efficiency was calculated using formula (6):6
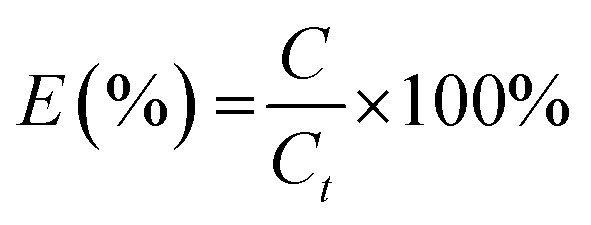
where *E* is the leaching efficiency (%), *C*_*t*_ is the total amount of substance in raw material (mg L^−1^), *C* is the amount of substance in leachate (mg L^−1^). The metal content (mg L^−1^) in the filtered solutions was determined using AAS.

The experimental data for each response showing leaching efficiency (Y_1_ – Li, Y_2_ – Co, Y_3_ – Ni, Y_4_ – Mn) were approximated by a second-order polynomial model:7
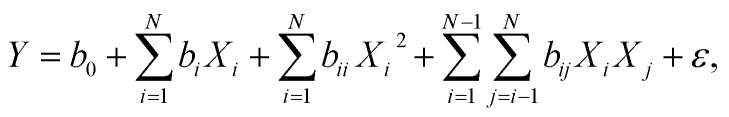
where *Y* – predicted value of the metal leaching efficiency response; *b*_0_ – intercept term; *X*_*i*_ and *X*_*j*_ – independent variables; *b*_*ii*_ and *b*_*ij*_ respectively – coefficients of quadratic and interaction terms; *ε* – error.^50^

Experimental design and subsequent regression analysis were performed using Design Expert 13 software. The statistical significance of the model as a whole and its individual terms were assessed using analysis of variance (ANOVA). Based on the calculated *F*-criterion for each model term, the *p*-value was determined, which was then compared with the significance level *α* = 0.05. The selection of the optimal model type (*e.g.*, linear or quadratic) was conducted based on sequential model sum of squares analysis. The quality of data approximation by the model was evaluated using the following indicators: coefficient of determination (*R*^2^), adjusted coefficient of determination (adjusted *R*^2^), and predicted coefficient of determination (predicted *R*^2^), as well as the “adequate precision” indicator (signal-to-noise ratio > 4) and coefficient of variation (CV, %). Model adequacy was confirmed by a statistically insignificant lack-of-fit criterion (lack-of-fit test), where *p* > 0.05 indicates that the model correctly describes the experimental data. Multi-criteria optimization was conducted using the desirability function. Within this approach, objectives for maximizing the leaching rate of target metals were formalized, while the controlling factors could vary within previously established ranges.

### 2.5 Investigation of leaching kinetics

To elucidate the mechanism of metal leaching from spent LIBs black mass, 13 models were studied (see Chapter 3.4.2 and Table S1–S3), from which three were selected that best describe the process (*R*^2^ > 0.85). The leaching progress was monitored by sampling at regular time intervals and analysing the metal content after filtration. Upon completion of the leaching experiments, the suspension was filtered, and the residue was dried overnight at 353 K. Based on the analysis of the leaching solution, the metal leaching efficiency was determined. In several series of experiments, mass balance was verified by calculating the metal content in the leaching solution and residues.

## 3 Results and discussion

The elemental composition of the electrode mass, determined by “wet chemistry” – AAS, presented in Table 4. Other metals, such as copper and iron, are present in smaller quantities (AAS analysis).

**Table 4:** Composition of the studied electrode mass

Element	Li	Co	Ni	Mn	Cu	Fe	C
wt%	6.55 ± 0.20	18.31 ± 0.55	11.11 ± 0.33	7.35 ± 0.22	0.51 ± 0.01	0.24 ± 0.01	55.92 ± 1.68

**Fig. 2:**
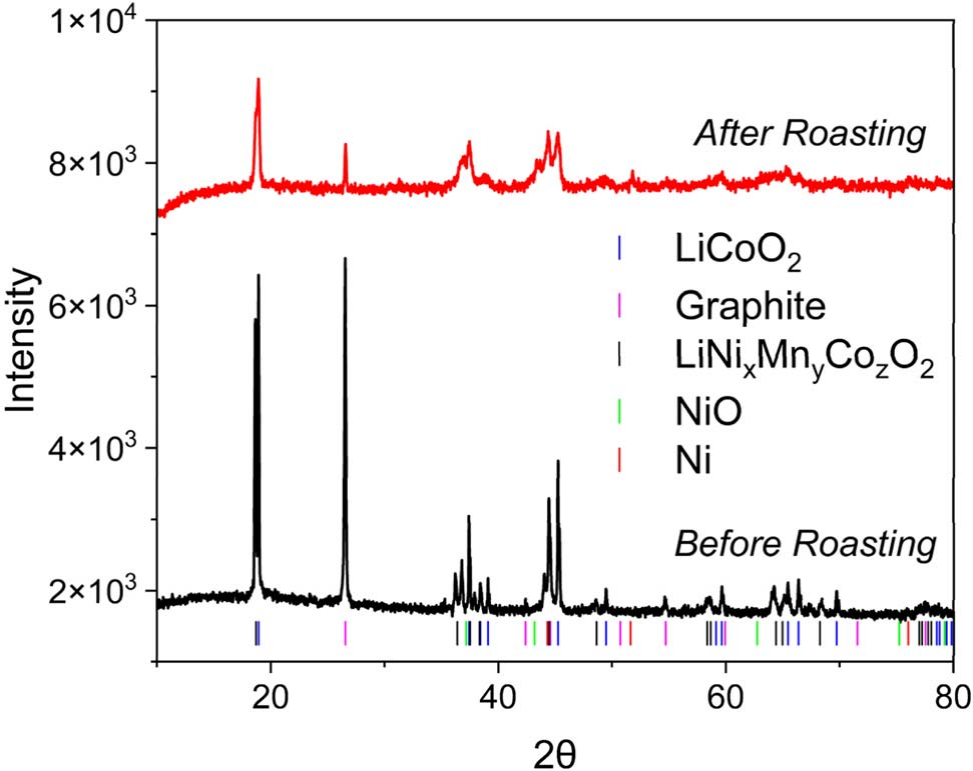
X-ray diffractograms of the studied black mass: before (before roasting) and after thermal treatment at 600 °C for 15 hours (after roasting).

To study the structural changes in the black mass caused by thermal treatment, XRD was employed. The results for the original and annealed samples are presented in Fig. 2. The analysis showed that the original electrode mass (before roasting) is a multiphase composite. Quantitative analysis by the Rietveld method revealed the presence of two main cathode phases: LiCoO_2_ (LCO, ∼18.4%) and LiCo_*x*_Ni_*y*_Mn_*z*_O_2_ (NMC, ∼18.1%). In addition to the active cathode components, graphite, which is the main anode material, is present in large quantities (∼60.6%). All identified crystalline phases are characterized by narrow and intense reflections, indicating a high degree of crystallinity of the original materials.

Following the annealing procedure applied to the <100 μm electrode mass fraction, thermal treatment at 600 °C for 15 hours resulted in significant phase and structural transformations in the composite (Fig. 2). Quantitative analysis by the Rietveld method revealed a significant change in phase composition. The dominant cathode phase after annealing remains LiCoO_2_, whose share increases to 49.6%. Part of the original NMC is preserved in an amount of 10.4%. At the same time, the appearance of new crystalline phases is observed: metallic nickel (20.2%) and nickel oxide (NiO, 4.8%), which indicates the reduction of nickel from its compounds under thermal exposure conditions. Graphite, the main anode material, is also preserved in a noticeable amount (15.1%), indicating its incomplete oxidation. In contrast to the original sample, the diffractogram of the annealed material shows noticeable broadening of all reflections and a general decrease in their intensity. This is a sign of structural degradation of the remaining phases, expressed in a decrease in crystallite size and accumulation of defects, despite the redistribution of phase composition.

As a result of annealing, the original crystalline composite based on graphite and layered oxides transformed into a new multiphase system characterized by the presence of metallic nickel and significant structural degradation.

### 3.1 Thermodynamic aspects of leaching with buffered citrate system in the presence of complexing agent

To understand the processes of metal recovery from cathode mass of spent LIBs by hydrometallurgical method, it is necessary to consider the thermodynamic aspects of dissolution, which are determined by the stability regions of various phases in aqueous solutions and are described by E_H_–pH diagrams (Pourbaix diagrams).^51,52^

**Fig. 3:**
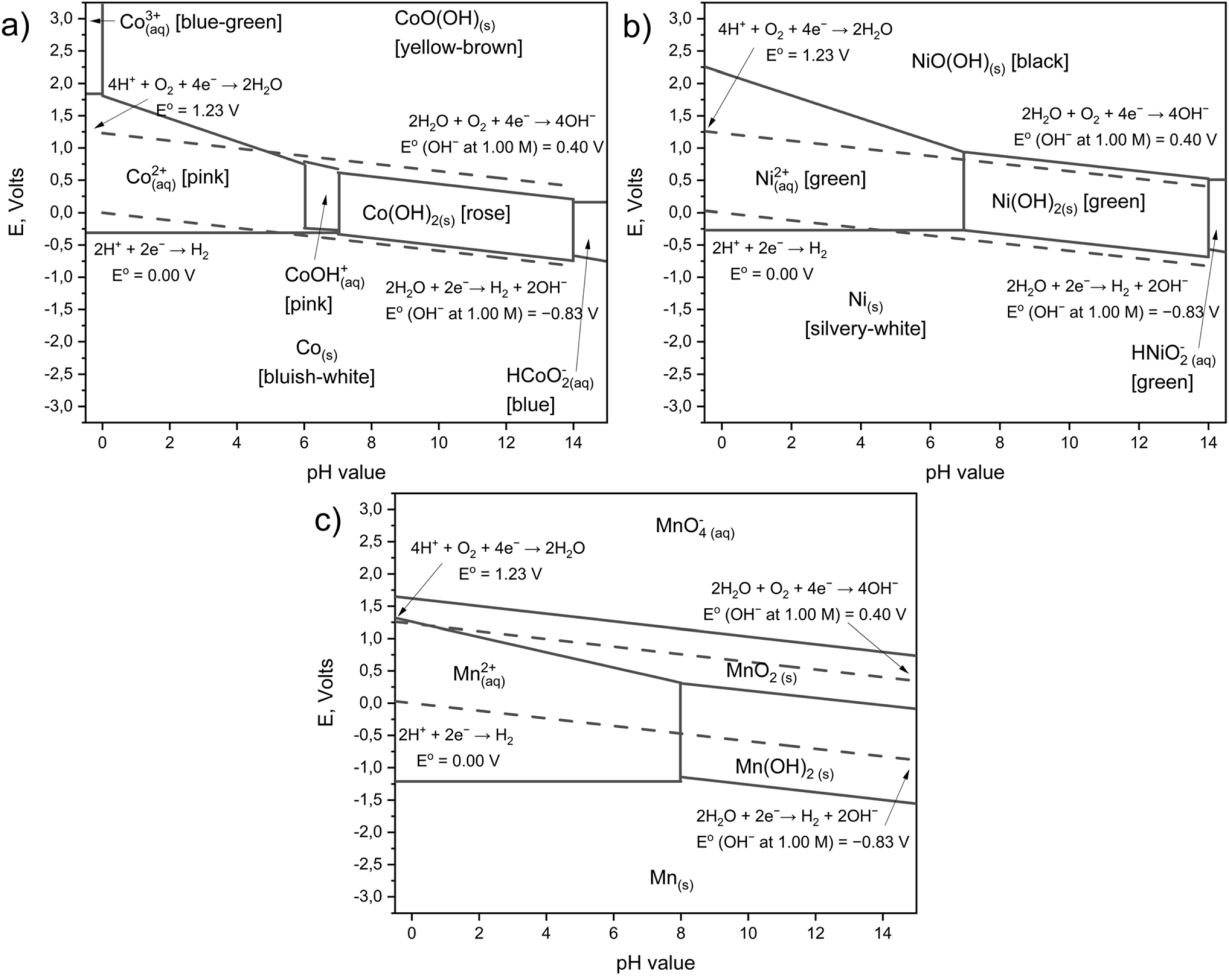
Pourbaix diagrams for the system (a) Co–H_2_O; (b) Ni–H_2_O and (c) Mn–H_2_O. During calculation, the concentration of metals Co; Ni and Mn are 0.1 mol L^−1^.^51^

As shown in the calculated E_H_–pH diagrams for Co–H_2_O, Ni–H_2_O and Mn–H_2_O systems, dissolution of high-valent oxide phases (LiCoO_2_, NiO(OH), MnO_2_ in LiCo_*x*_Mn_*y*_Ni_*z*_O_2_) in the absence of ligands requires extreme redox conditions.^51^ In particular, the transition of Co^3+^ into solution through Co^2+^ is impossible even in strong acid without reaching a potential of ∼+1.84 V, which lies above the water stability window, and Ni(iii)- and Mn(iv)-oxides also remain thermodynamically stable throughout the entire practical pH range (Fig. 3a–c).^51^

The introduction of a complexing agent transforms the system from a simple aqueous medium to one in which the metal predominantly exists as stable complexes with the ligand.^53,54^ This significantly increases the solubility of solid phases and facilitates the transfer of metals into solution, as well as broadens the range of conditions under which metal extraction can proceed without the need for extreme reductive potentials. A complete mathematical description of this process is provided in the SI (Section 2.1).

For real solutions, it is necessary to consider the contribution of chemical interactions in metal–ligand ionic equilibrium to the thermodynamic constant log *β*^Me^_T_. The main processes of chemical interaction are protonation/dissociation of ligand molecules depending on solution pH. Classical thermodynamics describes this through equilibrium constants: thermodynamic constant log *β*^Me^_T_, expressed through activities, concentration constant log *β*^Me^_C_, related to equilibrium concentrations, and conditional constant log *β*^Me^_L_(pH), accounting for side equilibria through *α*-coefficients. For practical calculations, conditional constants are most important since they reflect real solution conditions, considering ligand protonation, metal hydrolysis, and competing complexation reactions.^54^

**Fig. 4:**
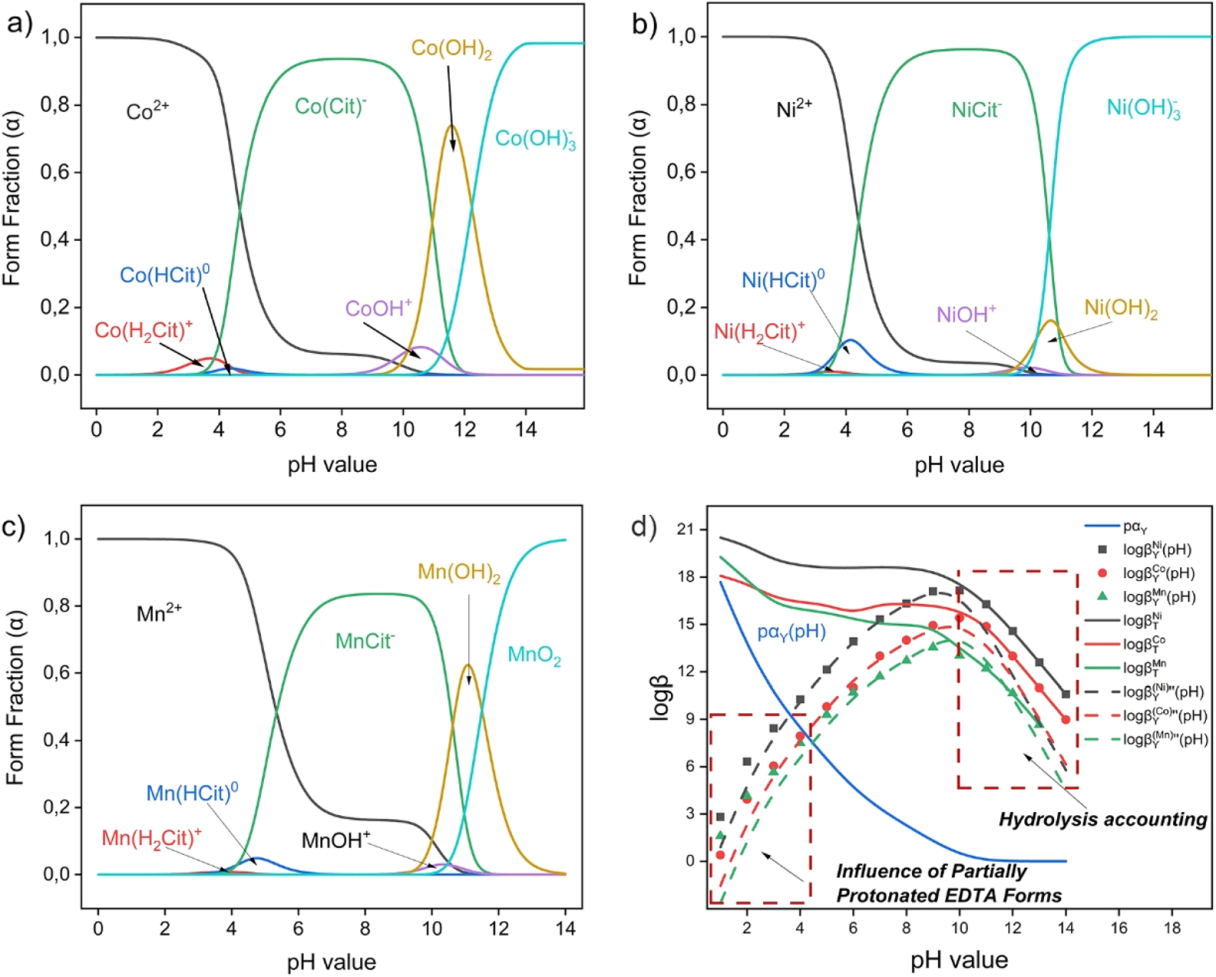
Distribution diagram of 1 : 1 Me-H_3_Cit systems (a) Co-H_3_Cit-H_2_O; (b) Ni-H_3_Cit-H_2_O and (c) Mn– H_3_Cit-H_2_O depending on solution pH. *C*_Me_ = 10^−4^ mol L^−1^; (d) thermodynamic, conditional and double conditional formation constants of Co-EDTA, Ni-EDTA and Mn-EDTA complexes depending on solution pH.

When considering the conditional complexation constant of metals log *β*^Me^_L_(pH) in the studied citrate buffer system, in the pH range 4–6 H_2_Cit^−^ and HCit^2−^ forms predominate in solution, which possess optimal buffer properties.^55^ Analysis of calculated distribution diagrams shows that in the specified pH range, citrate effectively forms soluble complexes with transition metal ions. For cobalt, Co(Cit)^+^ and Co(HCit)^0^ forms dominate (log *β*^Me^_H3Cit_(pH) = 4.62–4.86), for nickel – Ni(Cit)^+^ and Ni(HCit)^0^ (log *β*^Me^_H3Cit_(pH) = 5.09–5.29), and for manganese – Mn(Cit)^+^ и Mn(HCit)^0^ (log *β*^Me^_H3Cit_(pH) = 3.68–3.85) (Fig. 4a–c).^56,57^ These complexes function as intermediate forms that retain metals in solution after primary proton attack of the cathode solid phase and prevent their reprecipitation as hydroxides. The citrate buffer system provides pH stabilization in the optimal range, preventing its increase due to hydrolytic processes and proton consumption during oxide phase dissolution.^58^ This creates favorable kinetic conditions for subsequent binding of metals by a stronger chelating agent – ethylenediaminetetraacetic acid sodium salt (Na_2_EDTA).

EDTA is one of the most effective polydentate ligands for binding transition metal ions. However, realization of its high complexation potential critically depends on solution pH, which is due to the multistage nature of EDTA dissociation and the necessity of forming the active Y^4−^ form.^54^ The rationale for selecting EDTA is based on both thermodynamic principles and empirical stability constants. EDTA's hexadentate structure enables formation of 1 : 1 MeL_*n*_ complexes with six coordination bonds, effectively saturating the metal coordination sphere. The chelation effect results in favorable entropy changes (Δ*S* > 0) through displacement of solvent molecules, leading to negative Gibbs free energy (Δ*G* = Δ*H* − *T*Δ*S*) and thermodynamically stable complexes.^59^

Critically evaluated stability constants (log *K*_β_) demonstrate quantitative differences between EDTA and alternative ligands. EDTA complexes with Ni^2+^, Co^2+^, and Mn^2+^ exhibit stability constants 4–11 orders of magnitude higher than those of lower-denticity ligands. For instance, the Ni^2+^-EDTA complex (log *K*_β_ = 18.6) is ∼10^7^ times more stable than Ni^2+^-NTA (log *K*_β_ = 11.5). These higher stability constants enable more effective Me^2+^ solubilization in the leaching system.^59–61^

**Fig. 5:**
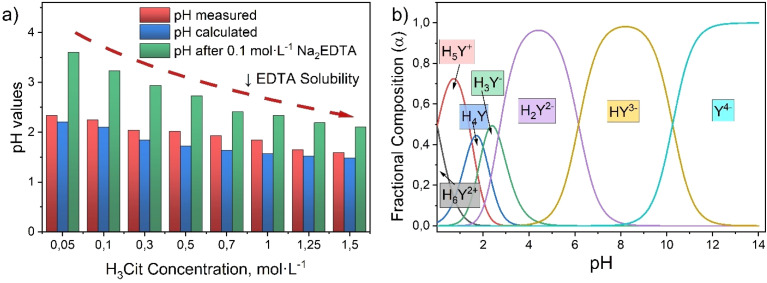
Effect of (a) H_3_Cit concentration and addition of 0.1 mol L^−1^ Na_2_EDTA on solution pH values; (b) distribution diagram of 0.1 mol L^−1^ Na_2_EDTA forms depending on solution pH.

Thermodynamic formation constants of EDTA complexes (log *β*^Me^_T_) for the studied metals constitute significant values: for Ni-EDTA – about 18.6, Co-EDTA – 16.3, Mn-EDTA – 13.8.^62^ However, these thermodynamic constants are realized only under conditions of complete ligand deprotonation, which in real conditions is achieved only at high pH values (Fig. 5, lines).

The conditional constant log *β*^Me^_Y_(pH) accounts for the fraction of active EDTA form through the function *pα*_Y_ = −log *α*_Y_ throu1gh the expression:

8 log *β*^Me^_Y_(pH) = log *β*^Me^_T_ − *pα*_Y_(pH)

At pH < 4, the fraction of Y^4−^ is negligibly small (*p*α_Y_>> 1), making complexation thermodynamically unfavorable despite having high thermodynamic constant values. Starting from pH ≈ 4–6, the value of *α*_Y_ increases sufficiently for conditional constants log *β*^Me^_Y_(pH) to remain double-digit for nickel and cobalt, ensuring effective binding of these metals (Fig. 5, points).

The double conditional constant 
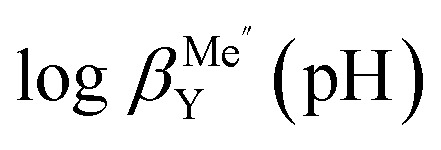
 additionally accounts for metal hydrolysis through the coefficient *α*_M_ through the expression:9
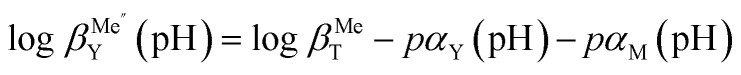


In the pH range 4–6, the contribution of hydrolysis is minimal (*pα*_M_ ≈ 0), which allows maintaining high values of effective stability constants. At pH > 10–12, a sharp decrease in observed constants is completely explained by an increase in the fraction of metal hydroxocomplexes, leading to competition between complexation and hydrolysis (Fig. 5, dashes).

The combination of citrate buffer and EDTA creates a synergistic effect, providing optimal thermodynamic conditions for metal leaching from NMC cathode materials. In the pH range 4–6, citrate functions as a “collector,” ensuring primary dissolution and retention of metals in solution, while EDTA acts as a “final acceptor,” binding metals into exceptionally stable chelate complexes.

Thermodynamic analysis shows that the selected pH range represents a compromise region where: (a) the fraction of active EDTA form (Y^4−^) is already sufficient for effective complexation; (b) metal hydrolysis remains minimal; (c) the citrate system provides effective buffering; (d) solubility of intermediate citrate complexes is maximal.

### 3.2 Effect of pH on Me leaching

Initial experiments contributed to the formulation of a new hypothesis regarding the use of a buffered citrate solution in conjunction with Na_2_EDTA. During the preparation of solutions with citric acid concentrations in the range of 0.05–1.5 mol L^−1^, it was found that the direct addition of EDTA to citric acid solutions resulted in partial or incomplete dissolution. This is due to the high acidity of the medium (pH < 2.5), characteristic of citric acid solutions of the aforementioned concentrations (Fig. 5a). Theoretical pH values of solutions were calculated according to the formula 
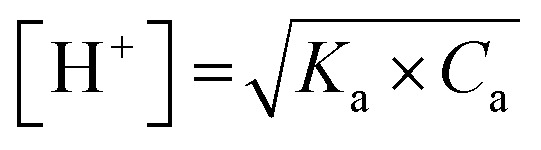
 , where *K*_a_ = 7.4 × 10^−4^ (p*K*_a_ = 3.13); *C*_a_ – citric acid concentration, mol L^−1^.

Theoretical assumptions about the decrease in Na_2_EDTA solubility in acidic solutions (pH < 3.00) confirmed experimentally observed phenomena (See Fig. S2). According to the distribution diagram of EDTA forms depending on solution pH (Fig. 5b), Na_2_EDTA transitions to protonated forms (H_4_Y; H_5_Y^+^; H_6_Y^2+^) (10):

10 H_6_Y^2+^ ⇌ H_5_Y^+^ ⇌ H_4_Y ⇌ H_3_Y^−^ ⇌ H_2_Y^2−^ ⇌ HY^3−^ ⇌ Y^4−^

which have water solubilities ≈ 5 g L^−1^ (Fig. S2), while the water solubility of H_2_Y^2−^ and Y^4−^ forms is 100 and 111 g L^−1^.^62^

For this reason, the successful application of EDTA in the leaching system requires creating conditions that ensure a more neutral or weakly acidic pH, at which EDTA exists in deprotonated, coordinatively active form (H_2_Y^2−^; HY^3−^). This served as the basis for transitioning to the next research stage, in which the solution composition was modified to stabilize pH by introducing a buffer system based on citric acid and sodium citrate.

The buffer system possesses resistance to pH fluctuations, making it particularly suitable for the leaching process involving a chelating agent.^54^ To select an appropriate solution composition, the Henderson–Hasselbalch eqn (11) was used:11
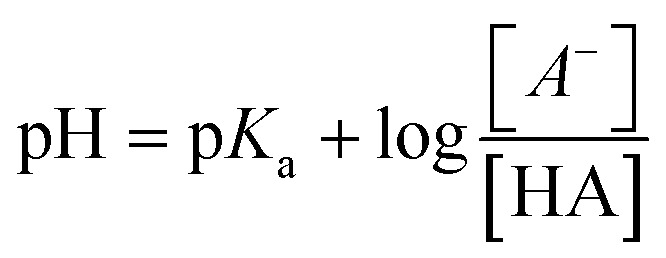
where [*A*^−^] is equilibrium concentration of the acid salt, mol L^−1^; [HA] is equilibrium concentration of the acid, mol L^−1^. Maintaining the specified pH value for a weak acid and its salt through the buffering mechanism, which consists of the constancy of the buffer system component ratio [*A*^−^] as an electron donor (Brønsted conjugate base) and [HA] an electron acceptor (Brønsted acid) upon dilution, promotes maintenance of complexation of metal ions by the chelating agent.

**Fig. 6:**
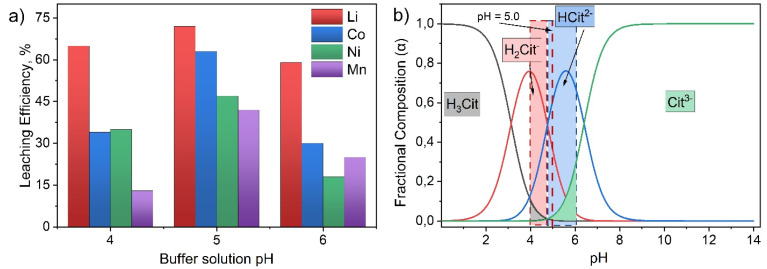
(a) Leaching efficiency of Li, Co, Ni and Mn depending on citrate buffer pH with 0.1 mol L^−1^ Na_2_EDTA; (b) distribution diagram of 0.1 mol L^−1^ citric acid forms depending on solution pH.

Based on calculations and thermodynamic justification in Chapter 3.1, citrate buffer solutions were prepared providing the following pH values 4.0; 5.0; 6.0, which served as working regions (Fig. 6b).

The results of electrode mass leaching in citrate buffer with addition of 0.1 mol L^−1^ Na_2_EDTA at 50 °C and S : L ratio = 20 g L^−1^ demonstrate a pronounced dependence of metal leaching efficiency on the buffer solution pH value (Fig. 6a). Maximum lithium leaching rate (72%) is achieved at pH = 5, which exceeds the indicators at pH = 4 and pH = 6 by 7% and 33%, respectively. For cobalt, a sharp decrease in efficiency is observed with increasing pH: from 63% at pH = 5 to 34% at pH = 4 and only 30% at pH = 6. Nickel leaching is characterized by intermediate values with an optimum of 47% at pH = 5. Manganese leaching efficiency varies from 13% (pH = 4) to maximum 42% (pH = 5), after which it decreases to 25% at pH = 6.

As noted in the thermodynamic aspects (Chapter 3.1), the fraction of dissociated EDTA forms (*α*_Y_) increases from pH = 4–5, leading to effective ion binding with double-digit complexation constants. This circumstance prevents the formation of insoluble Me forms, which are formed according to the reactions:

12 Co^2+^ + 2OH^−^ → Co(OH)_2_↓

13 Mn^2+^ + 2OH^−^+1/2O_2_ → MnO_2_↓ + H_2_O

14 Ni^2+^ + 2OH^−^ → Ni(OH)_2_↓

15 3Co(OH)_2_ + 1/2O_2_ → Co_3_O_4_ + 3H_2_O

16 Ni(OH)_2_ + OH^−^ → NiOOH + H_2_O

Secondly, the citrate buffer used in the system plays a dual role: it maintains a stable pH level during the leaching process and itself participates in complexation with metal ions during the initial stages of leaching.

According to the leaching results, it was established that the best metal leaching rates was observed at pH = 5.0, which allowed selecting this composition as optimal for further experiments involving Na_2_EDTA and the buffer system.

### 3.3 RSM optimization and factor effects on response functions

Application of RSM is a widely recognized approach for optimizing multifactor processes, including hydrometallurgical recovery of metals from spent lithium-ion batteries.^63,64^ Traditionally, studies focus on finding extreme values of response functions and evaluating the influence of independent factors (*A*, *B*, *C*, *etc.*) on individual dependent variables (Y_1_, Y_2_…Y_*i*_), which are considered statistically independent quantities.

#### 3.3.1 Statistical analysis

In the present study, the RSM-CCRD methodology was applied to a citrate buffer-based leaching system with Na_2_EDTA addition, with a fundamentally different purpose: evaluating the relationships between dependent variables (leaching efficiencies of Li, Co, Ni, and Mn) to identify leaching mechanisms. The crucial methodological principle is the hypothesis that statistical significance of factors and their interactions serves as a quantitative reflection of dominant chemical processes controlling the leaching of each metal.

Although response functions for different metals are mathematically modeled as independent variables within the RSM statistical framework, their actual behavior is determined by a complex set of interrelated processes, including ligand competition for coordination with metal ions, formation of passivating layers, and mutual influence on local pH values resulting from hydrolysis and complexation.^65–67^ Thus, statistically significant correlations and factor interactions in the RSM model reflect physicochemical patterns of the leaching process, allowing formulation of justified hypotheses about mechanisms that can subsequently be verified through kinetic studies.

The complete design matrix and experimentally obtained response function values are presented in Table S4. Based on experimental data, statistically significant quadratic models (*p* < 0.05) were constructed, whose characteristics are given in Table 5. Model adequacy is confirmed by model *F*- and *p*-values and by the absence of significant lack-of-fit (lack-of-fit *p* > 0.05) for all responses. The Adj-*R*^2^ – Pred-*R*^2^ < 0.2 criterion demonstrates consistency of fitting and predictive capability of the models. The signal-to-noise ratio (adeq. precision) substantially exceeds the threshold value of 4, and low coefficients of variation confirm high reproducibility of the experimental procedure.^64^ All models were reduced according to *p*-criterion with backward selection (alpha = 0.05).

**Table 5:** Summary metrics of model fitting

Response	Model *p*	Lack-of-fit *p*	*R* ^2^	Adj- *R*^2^	Pred- *R*^2^	Adeq. precision	CV, %	Model
Y_1_ (Li)	< 0.0001	0.8655	0.9528	0.9057	0.8398	17.10	2.07	Quadratic
Y_2_ (Co)	0.0007	0.8469	0.9182	0.8363	0.7172	13.7888	3.94	Quadratic
Y_3_ (Ni)	0.0001	0.6772	0.8649	0.7974	0.6643	11.2805	10.51	Quadratic
Y_4_ (Mn)	< 0.0003	0.5675	0.9329	0.8658	0.6704	13.0537	5.70	2FI

Based on the ANOVA test, the following final regression equations in coded variables were obtained, describing the response surfaces for leaching efficiency of each metal:

17 Y_1_ (Li) = 98.75 + 4.65*B* − 5.16*B*^2^

18 Y_2_ (Co) = 88.61 − 0.2449*A* + 6.49*B* + 0.2111*C* + 5.01*AB* − 4.60*BC* − 3.74*B*^2^

19 Y_3_ (Ni) = 50.41 + 2.29*A* + 8.41*B* + 4.57*C* + 4.50*AC* − 6.87*BC* − 3.41*B*^2^

**Fig. 7:**
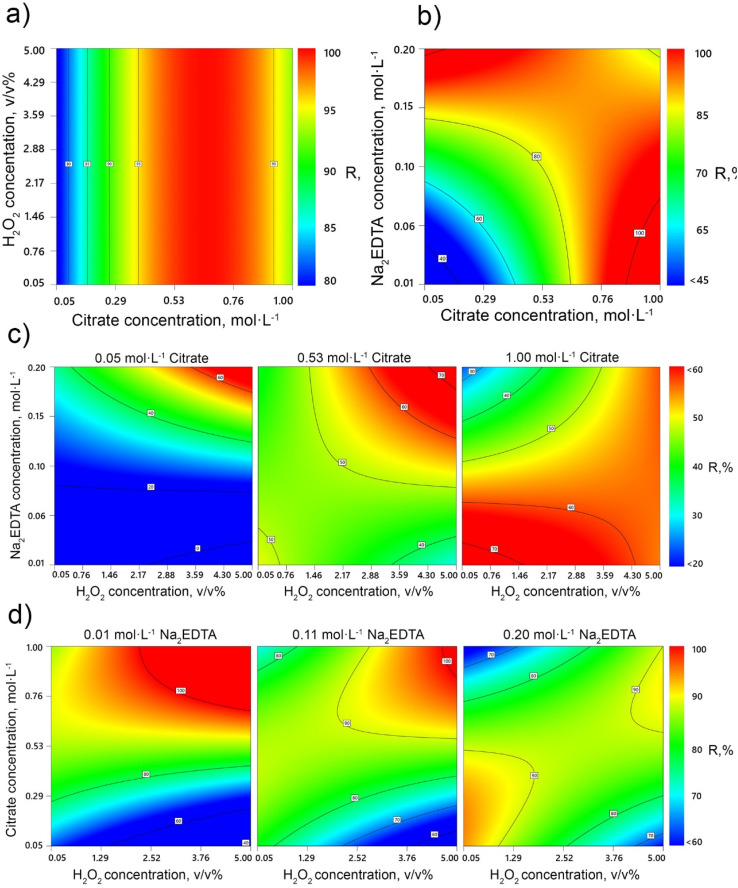
Response surface diagrams for metal leaching efficiency (*R*%) as a function of process parameters in the citrate buffer-H_2_O_2_-Na_2_EDTA leaching system: (a) Li; (b) Mn; (c) Co; (d) Ni.

20 Y_4_ (Mn) = 80.13 + 9.16*B* + 6.12*C* − 9.57*BC*

where *A*, *B*, and *C* are the coded values for the concentrations of H_2_O_2_, citrate buffer, and Na_2_EDTA, respectively. The response surfaces calculated according to these equations for each metal are shown in Fig. 7. Further analysis of these models allows us to identify chemical mechanisms controlling the leaching process.

#### 3.3.2 Analysis of factor effects and leaching mechanisms

##### 3.3.2.1 Effect of citrate buffer concentration (factor *B*)

Analysis of the regression models revealed the predominant influence of citrate buffer concentration (factor *B*) on the leaching efficiency of the metals studied. At the same time, the specific mechanisms of metal interaction with the citrate buffer show significant differences, reflecting the complex chemical nature of the process.

For the leaching of Li, Co, and Ni, a similar pattern is observed: the models contain both a statistically significant positive linear term (+4.65*B* for Li, +6.49*B* for Co, and +8.41*B* for Ni), as well as a significant negative quadratic term (for example, −5.16*B*^2^ for Li, −3.74*B*^2^ for Co, and −3.41*B*^2^ for Ni). This combination of coefficients clearly indicates the existence of an optimal citrate concentration.^68^ This effect is explained by the dual role of the buffer: on the one hand, increasing citrate concentration enhances the proton attack on the oxide matrix of the cathode material; on the other hand, excessively high concentrations lead to increased ionic strength and solution viscosity, ultimately impeding mass transfer.^38,69^ This conclusion is consistent with previous studies where an optimal acid concentration for leaching was identified. Thus, finding a balance between effective proton attack and minimizing negative rheological effects is the main challenge addressed by the RSM approach.

In the case of Mn, a strong positive linear effect is observed for both +9.16*B* and +6.12*C*, while the quadratic term for citrate is statistically insignificant. This indicates that, for manganese, the limiting factors are chelation (by both citrate and Na_2_EDTA) and proton attack, whereas negative mass transfer effects do not reach a critical threshold within the studied range.^38,69^

##### 3.3.2.2 Effect of H_2_O_2_ and Na_2_EDTA

In contrast to the citrate buffer, the individual linear effects of H_2_O_2_ (factor *A*) and Na_2_EDTA (factor *C*) are manifested more selectively.

The concentration of H_2_O_2_ as an independent linear factor is statistically insignificant for all four metals. This is explained by the fact that the primary function of H_2_O_2_ in this system is to reduce Co^3+^, Ni^3+^, and Mn^4+^ to their more soluble divalent forms at the initial stage.^70–72^ As soon as the Me^2+^ ion is formed at the phase boundary, it is immediately chelated by citrate or EDTA. According to Le Chatelier's principle, this binding removes the product (Me^2+^) from the reaction zone, lowering its concentration near the reaction interface and shifting the redox equilibrium toward the formation of soluble species. Thus, variation in H_2_O_2_ concentration only exerts an indirect influence on the overall process efficiency, and can be detected solely in synergistic interactions with other factors.

In contrast, the concentration of Na_2_EDTA demonstrates a more specific effect. For Li, its linear effect is statistically insignificant, as lithium is already present in its easily soluble ionic form, Li^+^. For cobalt, the linear effect of factor *C* is also insignificant. This is because, according to the model (eqn (18)), its extraction is primarily determined by the synergy of H_2_O_2_ and citrate, with the role of EDTA manifesting as a competitive interaction.

However, for nickel and manganese, the linear effect of factor *C* is statistically significant (+4.57*C* for Ni and +6.12*C* for Mn).^70,73^ This is due to the strong tendency of Ni^2+^ and Mn^2+^ to form passivating hydroxide layers (Ni_3_O_4_, MnO_2_) on particle surfaces. In this context, Na_2_EDTA plays a crucial role by immediately chelating these ions into exceptionally stable complexes, thereby preventing their hydrolysis and precipitation.

##### 3.3.2.3 Analysis of synergistic and competitive factor interactions

The statistical parameters quantitatively reflect the complex chemical relationships between the reagents, enabling the identification of both synergistic and competitive effects.

For cobalt and nickel, statistically significant positive interactions, *AB* (+5.01*AB*) and *AC* (+4.50*AC*), are observed, respectively. This synergistic effect corresponds to a two-step mechanism involving (a) reduction of Co^3+^ and Ni^3+^ to Co^2+^ and Ni^2+^ by hydrogen peroxide, and (b) immediate binding of Me^2+^ ions by chelating agents into stable complexes. This rapid stabilization shifts the reaction equilibrium towards complete dissolution, effectively suppressing reverse oxidation by dissolved O_2_ or hydrolysis of Me^2+^ ions, and manifests as a strong synergistic effect.^37,74^

Ligand competition and mass transfer limitations are evidenced by statistically significant negative *BC* interaction terms for Co, Ni, and Mn (−4.60*BC*, −6.87*BC*, and −9.57*BC*, respectively). The citrate-EDTA-H_2_O_2_ system implements a dual chelation mechanism. The citrate buffer (H_2_Cit^−^ and HCit^2−^) initiates proton attack on the cathode matrix, triggering dissolution and acting as a “collector”. At this intermediate stage, less stable but rapidly formed citrate complexes of Me^2+^ Me(Cit)^+^, Me(HCit)^0^ are produced, preventing immediate precipitation of hydrolysis products and maintaining high buffer capacity within the reaction zone. Thus, citrate anions serve as “carrier molecules” for metal ions.

Na_2_EDTA functions as a “final acceptor”, binding Me^2+^ into exceptionally stable Me-EDTA complexes. The high conditional stability constant (log *β*_Y_^pH^) for Me-EDTA complexes provides a thermodynamic driving force, irreversibly shifting the leaching equilibrium toward dissolution and preventing reoxidation or hydrolysis of Me^2+^ in bulk solution.^75,76^ However, this creates a kinetic bottleneck, slowing recomplexation and the transition to the thermodynamically stable Me-EDTA form, which appears in the model as a negative *BC* interaction.

#### 3.3.3 Multicriteria optimization and evaluation of mechanistic interactions

**Fig. 8:**
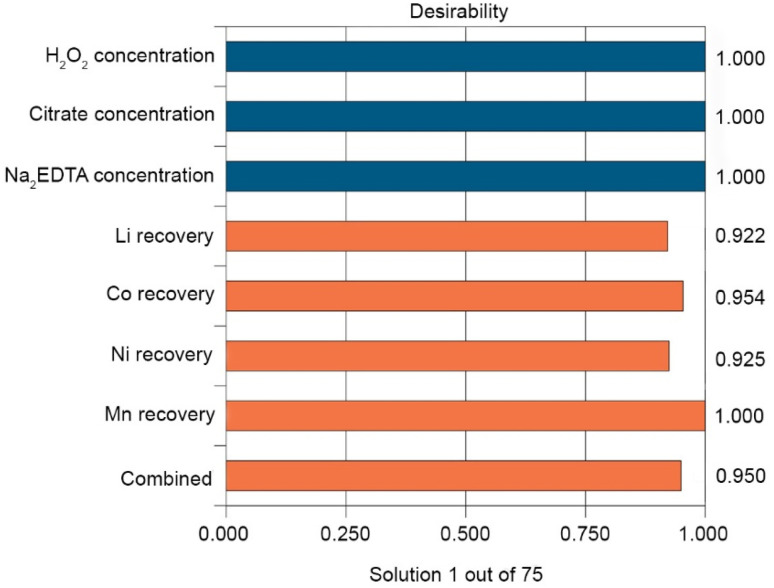
Desirability plot in optimal conditions.

To identify compromise optimal conditions that would enable high extraction rates for all target metals simultaneously, multicriteria optimization employing the desirability function was applied. As a result, the highest overall desirability (0.950) was reached under the following conditions: H_2_O_2_ concentration—2.425 v/v %, citrate buffer concentration—0.807 mol L^−1^, and Na_2_EDTA concentration—0.010 mol L^−1^ (Fig. 8).

The expected leaching efficiency values under these parameters are 98.23% for Li, 98.50% for Co, and 95.08% for Mn, while Ni remains the limiting component with a predicted leaching rate of 59.55%. Experimental validation under practical, economically feasible conditions (1.211 vol% H_2_O_2_, 0.778 mol L^−1^ citrate, and 0.010 mol L^−1^ Na_2_EDTA) confirmed the adequacy of the model, exhibiting results consistent with the prediction within a relative error of ±7%.

**Fig. 9:**
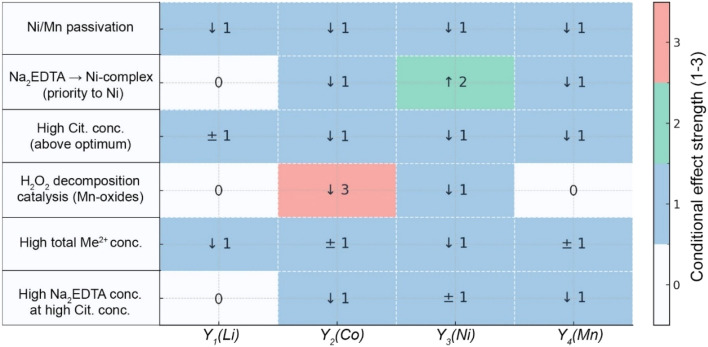
Cross-influence matrix for citrate buffer-EDTA-H_2_O_2_ leaching system (↑—acceleration/increase in the leaching efficiency of a given metal (Y_*i*_) under the action of the mechanism; ↓—deceleration/decrease in the leaching efficiency of Y_*i*_; ±—non-monotonic effect (there is an internal optimum/interval where the sign changes) or the effect is weak and unstable in terms of sign within the design area; Number 1–3—strength of the effect (point scale)).

The main mechanistic interactions demonstrated in Fig. 9 can be considered from the following positions: (1) surface passivation and mass transfer: formation of hydroxide layers Ni(OH)_2_/MnOOH reduces available surface area and local proton concentration, negatively affecting Li and Co leaching rates. This effect is minimized by maintaining redox balance for Ni (Na_2_EDTA + H_2_O_2_) and avoiding high combined concentrations of citrate and Na_2_EDTA for Mn (negative *BC* interaction). (2) Competitive chelation: EDTA complexation selectivity follows the series Ni^2+^ > Co^2+^ ≳ Mn^2+^, leading to free ligand redistribution and potentially reducing Co and Mn leaching rates when optimizing conditions for Ni. Similarly, competition for citrate ions between different cations reduces the buffer capacity of the system, which is critical for lithium delithiation. (3) Catalytic H_2_O_2_ decomposition: Mn-oxides exhibit peroxidase activity, reducing available H_2_O_2_ concentration and weakening synergistic effects *AB* (Co) and *AC* (Ni). This process requires monitoring of residual peroxide, especially at high manganese content in the initial material.

## 4 Investigation of electrode mass leaching kinetics

### 4.1 Effect of temperature on metal leaching kinetics from electrode mass

**Fig. 10:**
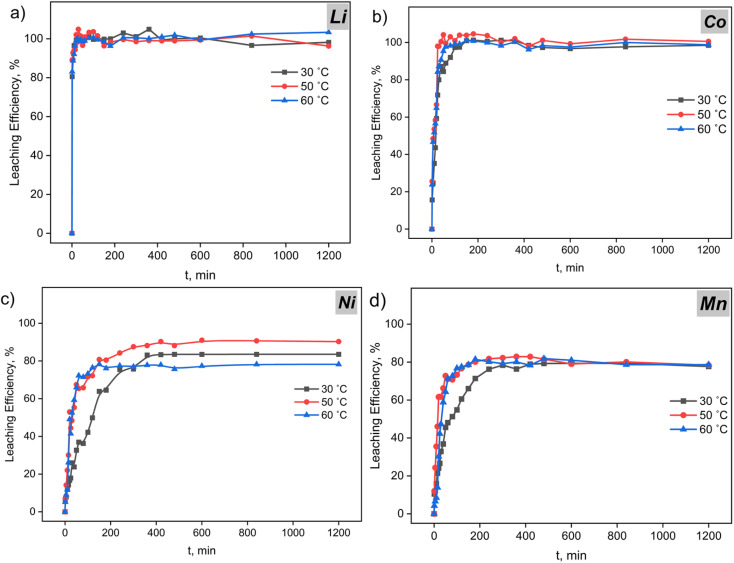
Kinetic curves of electrode mass leaching in citrate buffer with EDTA: (a) Li; (b) Co; (c) Ni; (d) Mn. Conditions: *C*(citrates) = 0.8 mol L^−1^; *C*(H_2_O_2_) = 1.2 v/v %; *C*(Na_2_EDTA) = 0.05 mol L^−1^; *v* = 400 rpm; S : L = 1 : 50.

Analysis of kinetic profiles (Fig. 10a–d) in the citrate–peroxide system with EDTA at pH ≈ 5 reveals the staged nature of the metal leaching process. Consequently, each metal undergoes three sequential regimes: short induction/surface activation (S-I) → reaction-controlled leaching growth (S-II) → deceleration due to product layer (S-III). The temperature optimum is reached at 50 °C, which is due to the balance between activation of chemical stages and competing passivation processes.

Lithium demonstrates exceptionally rapid leaching rate (*α* → 100% in 10–30 min) regardless of temperature regime. This is explained by the low-energy nature of cathode matrix delithiation and high solubility of lithium salts. Accordingly, temperature affects only the initial stage rate, while diffusion limitations do not manifest throughout the entire investigated range.^77^

Unlike lithium, cobalt achieves complete leaching efficiency (∼100%) only at temperatures of 50–60 °C. This behavior is associated with effective Co^3+^ → Co^2+^ reduction by H_2_O_2_ and subsequent stabilization as citrate and EDTA complexes. However, exceeding 50 °C does not provide additional benefit due to thermal decomposition of H_2_O_2_ and formation of a densified product layer.^78^

Nickel, in contrast, shows maximum sensitivity to temperature conditions. Optimal leaching efficiency is achieved at 50 °C, while at 60 °C, a critical reduction in efficiency is observed. Mechanistically, this is due to intensive consumption of the peroxide “redox resource” at elevated temperature, leading to local pH increase and formation of passivating Ni(OH)_2_ phases and basic citrates. Thus, the process transitions to diffusion control regime through the product layer.^79^

Manganese is characterized by the most pronounced passivation behavior among the studied metals. Despite acceleration of the initial stage with temperature increase, the leaching efficiency plateau remains limited to ∼75–85%. The crucial limiting factor is competition between reductive dissolution Mn(iv) → Mn(ii) and secondary oxidation of Mn^2+^ to poorly soluble MnO_2_·*x*H_2_O. Since the stability of Mn^2+^ complexes with citrate and EDTA is significantly inferior to analogous compounds of other metals, passivation manifests more prominently.^80^

### 4.2 Establishing the mechanism of metal leaching

For analysis of kinetic curve correspondence to models describing the mechanism of target metal leaching from cathode mass, 13 models were applied, whose classification can be seen in Table 6. Each model can be examined in more detail in the literature.^81–83^

**Table 6:** Kinetic models for the leaching process

Category	Model
Basic	First-order, Avrami–Erofeev, Peleg, Elovich
Diffusion	Parabolic, jander, Ginstling–Brounshtein
SCM	Reaction, diffusion, external diffusion
Modified	Fractional, Reich–Levenspiel, logarithmic

Based on preliminary approximation of 13 models for leaching kinetic curves in citrate buffer mixture with addition of complexing agent, 3 models with high correlation coefficients (*R*^2^) and logical suitability were selected (see SI).

Metal leaching from Li-ion battery cathode mass in citrate-EDTA medium follows a clearly expressed hierarchy of reactivity: Li ≫ Co > Ni > Mn. This sequence is determined by fundamental differences in the leaching mechanisms of each metal and forms the basis for understanding the kinetic patterns of the process.

#### 4.2.1 Lithium

**Fig. 11:**
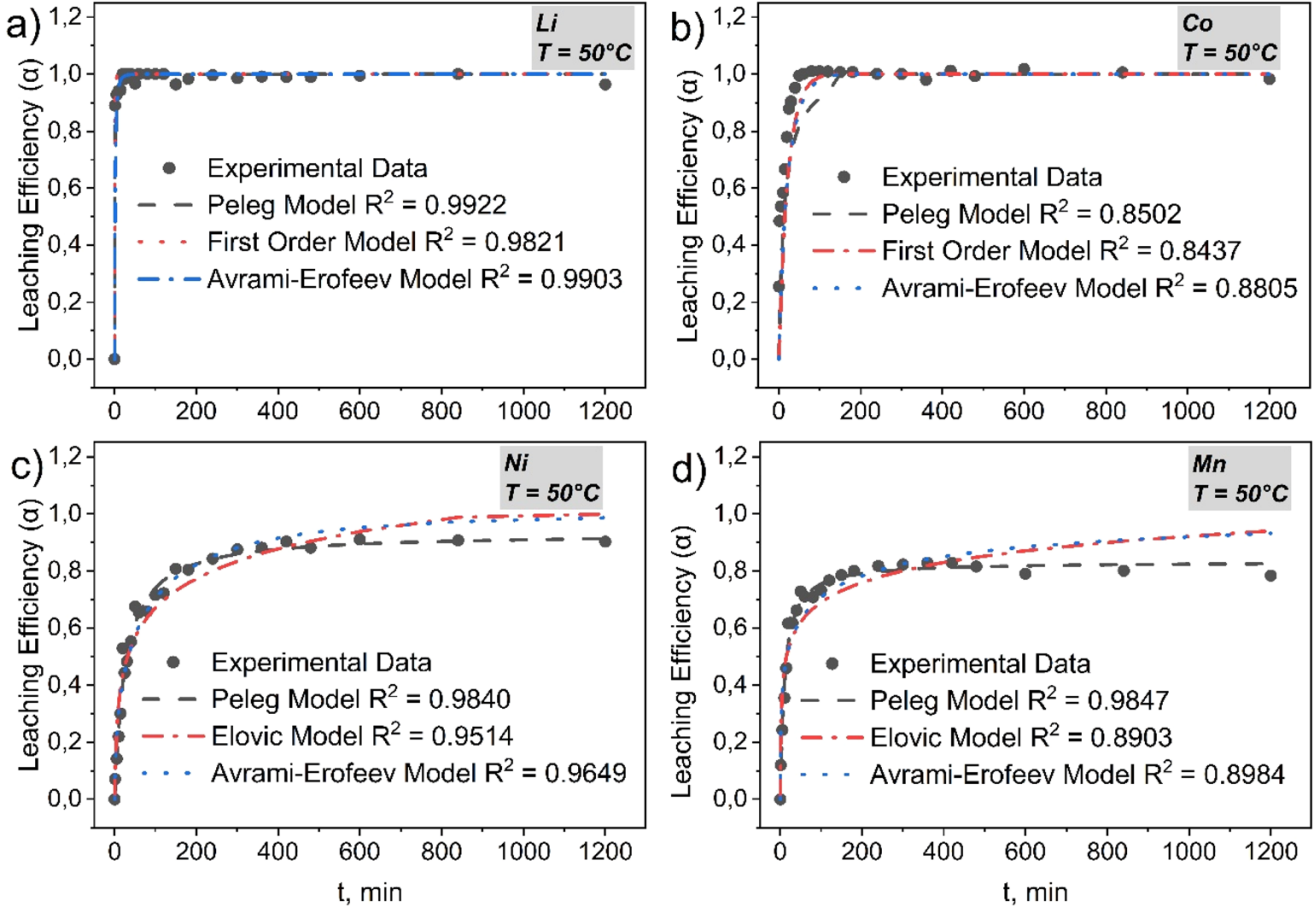
Kinetic curves of (a) Li, (b) Co, (c) Ni and (d) Mn at *T* = 50 °C. Conditions: *C*(citrates) = 0.8 mol L^−1^; *C*(H_2_O_2_) = 1.2 v/v %; *C*(Na_2_EDTA) = 0.05 mol L^−1^; *v* = 400 rpm; S : L = 1 : 50 (points – experimental data; dashes–model curves).

Lithium demonstrates exceptionally rapid leaching rate with *α* → 1 achieved within the first minutes regardless of temperature (Table S10). The superiority of the Avrami–Erofeev model (*R*^2^ = 0.9963 → 0.9903 → 0.9952 at 30 → 50 → 60 °C) over the first-order model (*R*^2^ = 0.9832 → 0.9821 → 0.9767) is explained by its ability to correctly describe the “explosive” initial section through stable small values of the exponent *n* = 0.297 → 0.265 → 0.238. The effective constant *k* demonstrates non-monotonic dependence (4.78 → 10 → 9.44), reaching maximum at 50 °C (Fig. 11a, S8a and S9a).

The Peleg model shows comparable quality (*R*^2^ = 0.9942 → 0.9922 → 0.9907) with characteristic change in initial rate *v*_o_ = 1/*k*_1_ (3.84 → 8.04 → 4.39 min^−1^), also passing through maximum at optimal temperature. Parameter *k*_2_ remains practically unchanged (∼1.01), confirming complete recovery at all temperatures (Table S10).

Mechanistically, the process corresponds to the “instantaneous delithiation → frontal completion” scenario without formation of a significant product layer. Consistently high approximation quality indicates dominance of surface-reaction control over diffusion processes throughout the entire temperature range.

#### 4.2.2 Cobalt

Cobalt is characterized by systematic superiority of the Avrami–Erofeev model (*R*^2^ = 0.976 → 0.881 → 0.957 at 30 → 50 → 60 °C) over first-order (*R*^2^ = 0.974 → 0.844 → 0.914) and Peleg models (*R*^2^ = 0.955 → 0.850 → 0.929) (Fig. 11b, S8b and S9b). A key feature is the pronounced nucleation and reaction front growth stage, which is reflected by the systematic decrease in exponent *n* = 0.883 → 0.691 → 0.668 with increasing temperature (Table S11). The effective constant *k* demonstrates characteristic non-monotonic dependence (0.046 → 0.091 → 0.077), reaching maximum at 50 °C and reflecting optimal balance between activation of Co^3+^ → Co^2+^ reduction and competing passivation processes.

The Peleg model shows corresponding decrease in *k*_1_ = 12.7 → 6.37 → 7.19, confirming acceleration of the initial stage up to optimal temperature. At elevated temperatures, accelerated H_2_O_2_ decomposition and local pH increase promote formation of basic salts, which explains the quality deterioration of all models at 50 °C (Table S11). This circumstance is demonstrated by XRD results of electrode mass during leaching time: even after 6 hours of leaching in citrate buffer system with Na_2_EDTA addition, the Co_3_O_4_ phase is present, blocking further leaching of cobalt, nickel and manganese (Fig. S10).

#### 4.2.3 Nickel

As shown in Fig. 11c, S8c and S9c, nickel demonstrates dominance of the Peleg model at moderate temperatures (*R*^2^ = 0.98 for 30–50 °C) with critical quality deterioration at 60 °C (*R*^2^ = 0.95). This behavior reflects a fundamental feature of nickel: monotonic increase in initial rate *v*_0_ = 0.0098 → 0.041 min^−1^ is accompanied by critical decrease in limiting leaching rate *α*_eq_ = 0.98 → 0.84 (Table S12).

Mechanistically, Ni^3+/4+^ → Ni^2+^ reduction determines initial activity, explaining the growth of kinetic parameters up to 50 °C. However, at 60 °C, accelerated H_2_O_2_ decomposition and local pH increase promote formation of passivating Ni(OH)_2_ phases and basic citrates. Product layer formation limits reagent diffusion, which is consistently recorded by the decrease in morphological parameter *n* in the Avrami–Erofeev model and reduction of *α*_eq_ in the Peleg model.

#### 4.2.4 Manganese

Manganese is characterized by exceptional consistency with the Peleg model (*R*^2^ = 0.987–0.943) and rigid limitation of the limiting leaching rate at *α*_eq_ ≈ 0.83–0.88 regardless of temperature (Fig. 11d, S8d and S9d). Initial rate reaches maximum at 50 °C, exceeding values at 30 °C and 60 °C by 4.5 and 2.5 times respectively, however high leaching efficiency (*α* > 0.90) remain unattainable at all investigated conditions (Table S13).

Mechanistically, manganese demonstrates the most pronounced passivation behavior. Rapid reductive dissolution Mn^4+^ → Mn^2+^ provides effective start, however weak complexation of Mn^2+^ with ligands intensifies competing processes: critical secondary oxidation of Mn^2+^ to poorly soluble MnO_2_·*x*H_2_O and formation of a diffusion barrier from passivating phases.

**Fig. 12:**
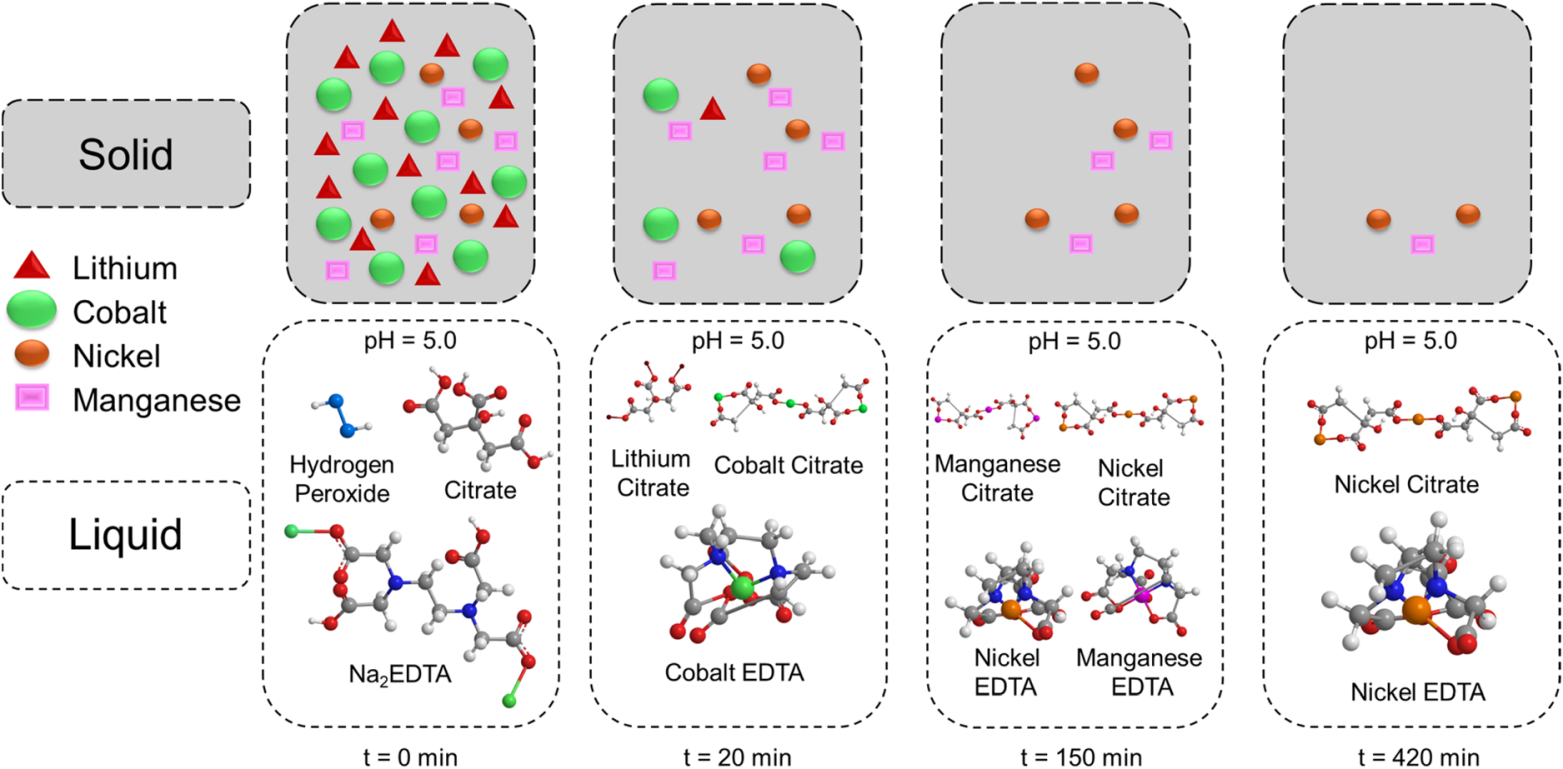
Scheme of the proposed leaching mechanism.

Leaching kinetics in the citrate-EDTA medium at pH ≈ 5, overall, follows a unified scenario schematized in Fig. 12. First, rapid matrix delithiation occurs: Li^+^ immediately goes into solution; simultaneously Co^3+^ is reduced to Co^2+^ and immediately stabilized by citrate and EDTA (Li-citrate and Co-EDTA complexes appear). Then, as the front advances, Ni and Mn are leached: their citrate and EDTA complexes are formed; simultaneously, however, passivating Ni(OH)_2_/MnO_2_·*x*H_2_O shells partially nucleate on particle surfaces, which limit the final leahing rate, primarily for Mn and, to a lesser extent, for Ni.

Models correlated with this mechanism give a consistent picture. For the “fast block” (Li, then Co), interfacial-reaction control dominates: curves are best described by Avrami–Erofeev (front growth, *n* < 1) and Peleg (high starting rate without “long tail”). For the “limited block” (Ni, Mn), separated parameters of the Peleg model are decisive: initial rate *v*_o_ increases with temperature, while limiting degree *α*_eq_ decreases due to passivation; in Avrami this manifests as a decrease in *n*. Ultimately, optimal temperature around 50 °C provides the best balance of “chemical activation ↔ minimal passivation”. Practically, therefore, maintaining pH ≈ 5, dosed H_2_O_2_ addition and enhanced Ni^2+^/Mn^2+^ chelation reduce the product layer barrier and increase final leaching efficiency.

Paying attention to the chemistry of the process, shown in the diagram in Fig. 12, it is necessary to note that based on the XRD and Rietveld analysis, the electrode mass after annealing consists predominantly of LiCoO_2_, LiCo_*x*_Ni_*y*_Mn_*z*_O_2_, metallic Ni, NiO, and graphite. The leaching mechanism thus proceeds *via* sequential dissolution and oxidation reactions, as summarized below: (i) LiCoO_2_ and LiCo_*x*_Ni_*y*_Mn_*z*_O_2_ undergo acid-promoted Li extraction and reduction of transition metals with subsequent complexation by citrate and EDTA; (ii) metallic Ni is oxidized by H_2_O_2_ and complexed; NiO dissolves in acidic conditions to form stable complexes; (iii) throughout the process, charge neutrality is maintained by lithium ion release, proton intercalation, and electron redistribution, with complex formation effectively preventing secondary precipitation. For detailed chemical reaction equations corresponding to each stage, see SI Section 3.1.1.

## 5 Thermodynamics of metal leaching processes

To quantitatively assess the energetic factors determining metal leaching efficiency, a comprehensive thermodynamic analysis was conducted using a two-level approach. Specifically, equilibrium state functions (Δ*H*, Δ*S*, Δ*G*) characterizing the overall thermodynamic possibility of dissolution and complexation processes were calculated, as well as transition state barrier parameters (Δ*H*^‡^, Δ*S*^‡^, Δ*G*^‡^) describing kinetic limitations of the rate-limiting stage. This approach allows for the separation of enthalpic and entropic contributions to the driving force and activation barrier, which is fundamentally important for understanding the process nature and optimising technological parameters.

Calculation of equilibrium parameters was performed using the van't Hoff equation based on temperature dependencies of equilibrium constants. Barrier parameters were determined from analysis of temperature dependencies of rate constants using the Eyring equation for first-order reactions, while activation energies for the Avrami model were calculated from corresponding constants of autocatalytic processes. Detailed results of thermodynamic analysis for each metal are presented in supplementary materials (See Chapter 4 in SI).

Comparison of thermodynamic parameters reveals fundamental patterns of leaching processes for different metals. Ranking by Gibbs barrier magnitude at 303 K shows the sequence: Co (92.1) > Ni (87.4) ≈ Mn (87.2) > Li (83.25) kJ mol^−1^, which corresponds to the upper limit for chemically controlled processes (∼40–100 kJ mol^−1^) (Tables S14–S17). These elevated activation energies arise from a complex multistage mechanism that encompasses both energy-intensive elementary steps and the formation of bulky chelate complexes. The process initiates with the cleavage of strong metal–oxygen bonds in the crystalline lattice and the crucial redox step—reduction of Co(iii), Ni(iii), and Mn(iv) ions by H_2_O_2_, which itself creates a substantial activation barrier. Subsequently, unlike simple systems, sequential formation of two distinct complex types occurs: initially, intermediate complexes with citrate ions form, followed by more stable terminal complexes with EDTA. The coordination of these polydentate ligands requires specific spatial orientation, which energetically hinders transition state formation. The dominant role of entropy represents a key factor: calculated extremely negative Δ*S*^‡^ values (ranging from −245 to −270 J mol^−1^ K^−1^) indicate that the transition state is highly organized and ordered. This implies that for the reaction to proceed, reagent molecules must converge in a low-probability configuration, creating a substantial entropic barrier (−*T*Δ*S*^‡^) that provides the primary contribution to the overall Gibbs activation energy (Δ*G*^‡^). Analysis of equilibrium thermodynamics reveals two fundamentally different types of behavior. Cobalt demonstrates entropy-determining equilibrium (Δ*S* > 0), where temperature increase enhances thermodynamic driving force. In contrast, nickel and manganese are characterized by entropy-limited processes with optimal conditions at moderate temperatures. Lithium occupies an intermediate position with relatively weak temperature sensitivity of equilibrium.

Analysis of equilibrium thermodynamics reveals two fundamentally different types of behavior. Cobalt demonstrates entropy-determining equilibrium (Δ*S* > 0), where temperature increase enhances thermodynamic driving force. In contrast, nickel and manganese are characterized by entropy-limited processes with optimal conditions at moderate temperatures. Lithium occupies an intermediate position with a relatively weak temperature sensitivity of equilibrium.

Correlation with kinetic models confirms the physicochemical validity of thermodynamic analysis. Preferential description of lithium and cobalt by the Avrami–Erofeev model is consistent with their relatively low activation energies for this model. Dominance of the Peleg model for nickel and manganese correlates with their increased activation energies and indicates a significant role of mass transfer limitations in the overall leaching mechanism.

## 6 Comparison of leaching efficiency with other systems

**Fig. 13:**
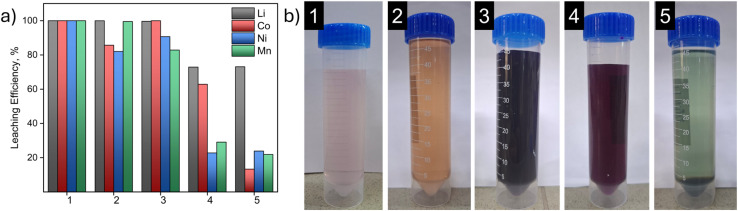
(a) comparative diagram of metal leaching efficiency; (b) comparative photographs of leached liquor for different systems: (1) 2.0 mol L^−1^ H_2_SO_4_ + 5 v/v % H_2_O_2_, *T* = 70 °C; (2) 1.25 mol L^−1^ H_3_Cit + 1 v/v % H_2_O_2_; *T* = 90 °C; (3) 0.8 mol L^−1^ citrate buffer + 1.211 v/v % H_2_O_2_ + 0.05 mol L^−1^ Na_2_EDTA, pH = 5.0, *T* = 50 °C; (4) 0.8 mol L^−1^ citrate buffer + 0.05 mol L^−1^ Na_2_EDTA, pH = 5.0, *T* = 50 °C (5) 0.8 mol L^−1^ citrate buffer + 1.211 v/v % H_2_O_2_, pH = 5.0, *T* = 50 °C.

To determine the competitiveness of the developed reagent system, a comparative analysis was conducted with traditional inorganic and organic leaching agents. Results of experimental investigation of metal leaching efficiencies are presented in Fig. 13.

Data analysis reveals that the developed system (mode 3) demonstrates competitiveness compared to traditional approaches. Specifically, the inorganic system based on sulfuric acid (mode 1) provides quantitative leaching of all metals, but requires harsh conditions (high acidity, temperature 70 °C) and is characterized by significant corrosion risks. The organic system based on citric acid (mode 2) requires even higher temperature (90 °C) and shows reduced efficiency for cobalt and nickel.

The developed reagent system (mode 3) provides high leaching efficiencies: Li—100%, Co—98.65%, Ni—90.69%, Mn—82.87% under moderate conditions (50 °C, pH 5). The key advantage is the synergistic effect of three components: H_2_O_2_ provides oxidative destruction of cathode materials, citrate buffer maintains stable pH, and Na_2_EDTA prevents metal reprecipitation through formation of stable complexes.

Comparison with incomplete systems (modes 4 and 5) confirms the critical importance of each component. The absence of H_2_O_2_ (mode 4) leads to a sharp reduction in leaching due to the preservation of the structural integrity of the materials. The absence of Na_2_EDTA (mode 5) causes the cobalt leaching efficiency to drop to 13.35% due to hydrolytic losses, as well as precipitate formation, most likely due to the precipitation of Co_3_O_4_, Ni_3_O_4_, and MnO_2_ (Fig. 13b), and also coprecipitation with Fe(OH)_3_.

## 7 Conclusion

This work considers a new approach to reagent leaching regimes based on citrate buffer with Na_2_EDTA for recovery of critical metals from spent lithium-ion batteries' black mass.

For the first time, comprehensive thermodynamic analysis of the Co–Ni–Mn–Li-citrate-EDTA-H_2_O system was conducted with calculation of Pourbaix diagrams and the calculation of conditional complexation constants. It was established that the synergistic effect of dual chelation ensures metal stabilization in solution through proton attack and sequential formation of citrate complexes (intermediate stage) with subsequent transition to highly stable Me-EDTA complexes (final stage). The pH range 4.0–6.0 represents an optimal region where the fraction of active Na_2_EDTA forms is sufficient for effective complexation with minimal metal hydrolysis.

Application of response surface methodology with central composite rotatable design enabled identification of optimal parameters for the leaching process reagent regime under the following conditions: 1.211 v/v% H_2_O_2_, 0.778 M citrate buffer, and 0.05 M Na_2_EDTA. Under these conditions, high leaching efficiencies were achieved: Li—100.0%, Co—98.65%, Ni—90.69%, and Mn—82.87% within 2–4 hours, which exceeds the performance of traditional methods under significantly milder process conditions (*T* = 50 °C). Response surface methodology provided quantitative assessment of factor interactions, revealing complex relationships in the multicomponent system. Significant positive synergies were established: H_2_O_2_-citrate for cobalt leaching and H_2_O_2_-EDTA for nickel leaching. These statistical interactions serve as quantitative reflections of underlying chemical processes, enabling application of scientifically-based optimization strategies.

Application of kinetic models revealed fundamental differences in the leaching mechanisms of individual metals. Lithium and cobalt are characterized by rapid kinetics with interfacial-reaction control, best described by Avrami–Erofeev and Peleg models. Nickel and manganese demonstrate diffusion-limited behavior with passivating layer formation, optimally described by the Peleg model. Thermodynamic analysis of activation barriers confirmed the energy barrier sequence: Co (92.1)> Ni (87.4) ≈ Mn (87.2)> Li (83.25) kJ mol^−1^.

## Author contributions

Saken Abdimomyn: writing – review & editing, writing – original draft, methodology, investigation, conceptualization. Zhulduz Zhanatkyzy: methodology, investigation. Grigoryev Artur: writing – original draft, methodology, investigation. Seilbek Malik: methodology, investigation. Kayirgali Zhumadil: methodology, investigation. Sergey Nechipurenko: methodology, investigation. Fyodor Malchik: writing – review & editing, supervision, conceptualization, project administration, funding acquisition.

## Conflicts of interest

The authors declare that they have no known competing financial interests or personal relationships that could have appeared to influence the work reported in this paper.

## Supplementary Material

RA-015-D5RA06978E-s001

RA-015-D5RA06978E-s002

RA-015-D5RA06978E-s003

## Data Availability

The data supporting this article have been included as part of the supplementary Information (SI). Supplementary information: detailed experimental procedures, raw data from the leaching experiments, results of kinetic and thermodynamic modeling, and comprehensive statistical analysis related to the optimization of the metal recovery process; figures illustrating the experimental setup, kinetic curves, statistical model diagnostics, and tables with correlation coefficients, ANOVA results, and calculated thermodynamic functions. See DOI: https://doi.org/10.1039/d5ra06978e.
