# Supplementary material for: Citrate-EDTA-H2O2 buffering leaching solution for Ni/Co/Mn recovery from spent lithium-ion battery black mass
Source: RSC Adv. 2025 Oct 27;15(48):40864–82. doi: 10.1039/d5ra06978e (PMC12557716; doi:10.1039/d5ra06978e)
Supplement: RA-015-D5RA06978E-s001 [file RA-015-D5RA06978E-s001.pdf]

Supplementary Information (SI) for RSC Advances.
This journal is © The Royal Society of Chemistry 2025

Citrate-EDTA-H_2O_2 Buffering Leaching Solution for Ni/Co/Mn Recovery From Spent Lithium-ion Battery Black Mass

*Saken Abdimomyn, Zhulduz Zhanatkyzy, Grigoryev Artur, Seilbek Malik, Kayirgali Zhumadil, Sergey Nechipurenko, Fyodor Malchik**

Faculty of Chemistry and Chemical Technology,
Al-Farabi Kazakh National University,
Al-Farabi 71/23, Almaty, Kazakhstan

**Primary corresponding author: Fyodor Malchik*
E-mail address: frodo-007@mail.ru

Full name	Information
Saken Abdimomyn	abdimomyn03@gmail.com *Researcher ID: GOW-8420-2022, ORCID: 0000-0002-5985-9050, Scopus ID: 57518892100, https://orcid.org/my-orcid?orcid=0000-0002-5985-9050*
Zhuldyz Zhanatkyzy	*zhuldyzjjj@mail.ru ResearcherID:OBN-6937-2025, ORCID: 0009-0005-8542-0622, Scopus ID: _ https://orcid.org/0009-0005-8542-0622*
Grigoryev Artur	*artur.grigoryev8@gmail.com Researcher ID: MDS-5070-2025, ORCID: 0009-0004-0532-4693, https://orcid.org/my-orcid?orcid=0009-0004-0532-4693*
Seilbek Malik	*seilbekmalik@gmail.com Researcher ID: OEO-7602-2025, Scopus ID: 59399902900, ORCID: 0009-0000-0662-6950 https://orcid.org/0009-0000-0662-6950*
Kayirgali Zhumadil	*maldybayevkaiyrgali@gmail.com Scopus Author ID: 57470372700, ORCID: 0000-0003-2752-4720, https://orcid.org/0000-0003-2752-4720*
Sergey Nechipurenko	*nechipurenkos@mail.ru ORCID: https://orcid.org/0000-0002-7463-1679*
Fyodor Malchik	*frodo-007@mail.ru ResearcherID: D-5721-2015, ORCID: 0000-0001-6381-0738, Scopus Author ID: 57196147903, https://orcid.org/0000-0001-6381-0738*

Citrate-EDTA-H_2O_2 Buffering Leaching Solution for Ni/Co/Mn Recovery From Spent Lithium-ion Battery Black Mass

*Saken Abdimomyn, Zhulduz Zhanatkyzy, Grigoryev Artur, Seilbek Malik, Kayirgali Zhumadil, Sergey Nechipurenko, Fyodor Malchik**

Faculty of Chemistry and Chemical Technology,
Al-Farabi Kazakh National University,
Al-Farabi 71/23, Almaty, Kazakhstan

**Primary corresponding author: Fyodor Malchik*
E-mail address: frodo-007@mail.ru

SUPPORTING MATERIALS

1 EXPERIMENTAL PART

1.1 Preparation of the studied electrode mass material

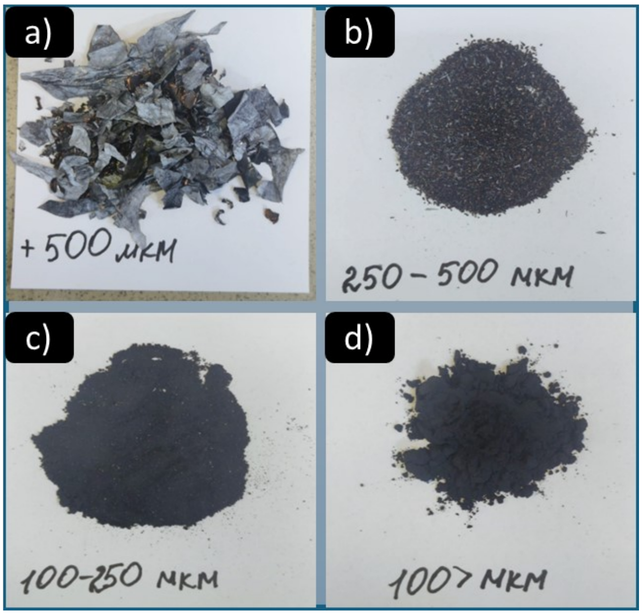

Figure S1. LIB fractions after separation: a) 500-1000 µm b) 250-500 µm c) 100-250 µm; d) <100 µm

1.2 Investigation of leaching kinetics

Table S1. Correlation coefficients of models used for cobalt leaching kinetics

**Cobalt**	R^2		
**Model**	**30 °C**	**50 °C**	**60 °C**
**Avrami–Erofeev**	0,976	0,881	0,957
Elovich	0,822	0,759	0,811
**First-order**	0,974	0,844	0,914
Fractional-order (n≈2.00)	0,713	0,658	0,709
Ginstling–Brounshtein	0,442	0,312	0,243
Jander	0,492	0,312	0,243
Logarithmic	0,374	0,358	0,390
Parabolic diffusion	-0,864	-1,595	-2,157
**Peleg**	0,955	0,850	0,929

Reich–Levenspiel (m≈2.00)	0,774	0,844	0,814
SCM (ash/product diffusion)	0,510	0,419	0,269
SCM (film diffusion)	0,419	0,322	0,231
SCM (surface reaction)	0,442	0,312	0,243

Table S2. Correlation coefficients of models used for nickel leaching kinetics

**Nickel**	R^2		
**Model**	**30 °C**	**50 °C**	**60 °C**
**Avrami–Erofeev**	0,958	0,930	0,785
**Elovich**	0,888	0,935	0,816
First-order	0,688	0,628	0,337
Fractional-order (n≈2.00)	0,742	0,760	0,406
Ginstling–Brounshtein	0,712	0,639	0,377
Jander	0,722	0,683	0,384
Logarithmic	0,888	0,935	0,816
Parabolic diffusion	0,851	0,689	0,525
**Peleg**	0,991	0,999	0,998
Reich–Levenspiel (m≈2.00)	0,742	0,760	0,406
SCM (ash/product diffusion)	0,712	0,639	0,377
SCM (film diffusion)	0,597	0,413	0,274
SCM (surface reaction)	0,664	0,555	0,322

Table S3. Correlation coefficients of models used for manganese leaching kinetics

Mn	R^2		
**Model**	**30 °C**	**50 °C**	**60 °C**
**Avrami–Erofeev**	0,939167	0,810265	0,797936
**Elovich**	0,916777	0,83803	0,813909
First-order	0,578958	0,314797	0,332414
Fractional-order (n≈2.00)	0,635431	0,35487	0,372843
Ginstling–Brounshtein	0,613134	0,343016	0,366995
Jander	0,620912	0,347502	0,363931
Logarithmic	0,916777	0,83803	0,813909
Parabolic diffusion	0,770708	0,481774	0,54788
**Peleg**	0,997559	0,999105	0,994177
Reich–Levenspiel (m≈2.00)	0,635431	0,35487	0,372843
SCM (ash/product diffusion)	0,613134	0,343016	0,366995
SCM (film diffusion)	0,488417	0,235384	0,288094
SCM (surface reaction)	0,553221	0,29128	0,32722

2. RESULTS AND DISCUSSION

2.1 Thermodynamic aspects of leaching with buffered citrate system in the presence of complexing agent

Introduction of a complexing agent L into the system shifts the description from a "pure" M–H_2O system to an Me–L–H_2O, system, in which the main fraction of dissolved metal is present as MeL_n complexes [53,54]. The decrease in free cation activity $a(Me^{z+})$ leads to two fundamental consequences:

i) Increase in apparent solubility of solid phases. For the reaction

$$Me(OH)_2(s) \rightleftharpoons Me^{2+} + 2OH^- , K_s = a(Me^{2+}) \cdot a(OH^-)^2 \quad (1)$$

where $a(Me^{2+})$ и $a(OH^-)^2$ are the activities of metal ion and hydroxide, respectively, in the presence of ligand L, the total (dissolved) metal concentration is expressed through the "binding polynomial" *Φ*:

$$[Me]_{tot} = [Me^{z+}]_{free}\, \Phi \quad (2)$$

$$\Phi = 1 + \sum_{n \geq 1} \beta_n \alpha_n^{(L)} [L]_{free}^n + \sum_{i \geq 1} \beta_{OH,i} [OH^-]^i + \ldots \quad (3)$$

where β_n are the overall formation constants for Me + nL ⇌ MeL_n; $\alpha_n^{(L)}$ are the fraction coefficients accounting for ligand protonation at given pH; $\beta_{OH,i}$ are the formation constants of hydroxo forms $Me(OH)_i$. Then the "apparent" solubility constant equals:

$$K_s^{app} = [Me]_{tot} a(associated\ ions)^{\vartheta} = K_s \cdot \Phi \quad (4)$$

Since $\Phi \geq 1$, always $K_s^{app} \geq K_s$, i.e., the "effective" solubility of the solid phase increases and the probability of metal transition to soluble Me^{z+} form is high.

*ii) Shift of redox boundaries on the Pourbaix diagram***.** For the Me_{Ox}/Me_{Red} pair, the effective potential ($E^{'}$) is determined by the following equition:

$$E^{'} = E^0 + \frac{RT}{nF} ln(\frac{a(Me_{Ox})}{a(Me_{Red})}) \quad (5)$$

$E^{'}$ decreases if $a(Me_{Red})$ exhibits higher complex stability constants ($\beta_{Red} \gg \beta_{Ox}$) and/or if pH increases so that the fraction of deprotonated ligand form grows. This expands the region of thermodynamic stability of soluble Me (II) forms and thereby facilitates leaching without extreme E_h.

2.2 Effect of pH on Me leaching

During preparation of citric acid solutions in the concentration range of 0.05 – 1.5 M with Na_2EDTA addition, it was found that at pH < 3, EDTA precipitates due to molecular transition to protonated form and decreased solubility to 5 g/L. The figure shows a photograph of the precipitate on filter paper after adding Na_2EDTA to 1.5 M citric acid solution and attempting dissolution at T = 50°C for 2 hours. The precipitate appears as a white suspension of amorphous type.

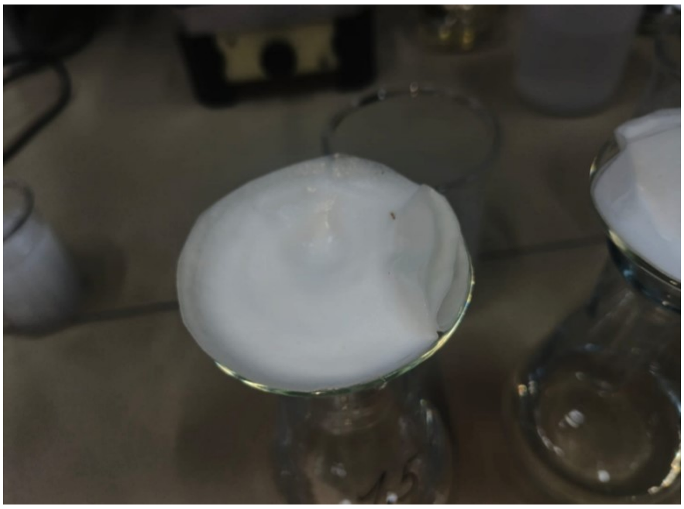

Figure S2. H_4EDTA precipitates at pH < 2.00

2.3 RSM optimization and factor effects on response functions

2.3.1 Statistical analysis

Table S4. Experimental design matrix and observed results

Run	Std	Factors			Response functions					pH initial	pH after leaching
		X_1	X_2	X_3	Y_1 (Li)	Y_2 (Co)	Y_3 (Ni)	Y_4 (Mn)	Y_5 (Reagent's Price)	5,075	5,82
		v/v %	M	M	%	%	%	%	USD	5,002	6,782
1	4	4.00	0.81	0.05	100.00	100.00	53.78	90.49	0.89	5,066	5,584
2	6	4.00	0.24	0.16	87.35	80.02	62.60	83.77	0.68	5,056	8,803
3	3	1.05	0.81	0.05	100.00	91.03	55.82	89.15	0.83	5,13	6,209
4	11	2.53	0.05	0.11	77.45	65.72	24.41	60.04	0.36	5,119	6,441
5	18	2.53	0.53	0.11	100.00	82.99	50.84	88.09	0.77	5,015	8,343
6	15	2.53	0.53	0.11	94.35	90.01	60.54	81.32	0.77	5,106	5,89
7	2	4.00	0.24	0.05	88.30	67.89	23.32	48.85	0.41	5,092	5,803
8	10	5.00	0.53	0.11	100.00	88.08	52.90	90.39	0.82	5,02	5,494
9	14	2.53	0.53	0.2	98.67	88.23	51.88	89.92	0.99	5,009	5,601
10	8	4.00	0.81	0.16	100.00	93.77	60.37	92.47	1.16	5,012	5,51
11	12	2.53	1	0.11	90.15	87.00	55.91	86.48	1.16	5,058	6,756
12	7	1.05	0.81	0.16	96.15	84.72	50.25	79.83	1.09	5,006	8,363
13	19	2.53	0.53	0.11	97.89	85.65	47.32	78.24	0.77	5,091	6,645
14	1	1.05	0.24	0.05	89.67	79.46	29.45	55.43	0.35	5,084	5,82
15	16	2.53	0.53	0.11	100.00	84.66	46.39	82.16	0.77	5,071	8,158
16	9	0.05	0.53	0.11	97.30	87.11	44.16	77.18	0.72	5	6,348
17	13	2.53	0.53	0.01	100.00	92.5721	48.6694	77.4098	0.52	5,067	6,043
18	5	1.05	0.24	0.16	88.4106	90.9193	45.6342	87.9807	0.61	5,075	5,82
19	17	2.53	0.53	0.11	100.00	93.2032	51.4975	89.4911	0.77	5,002	6,782

2.3.2 Factor effects on Li leaching

Table S5. The analysis of variance (ANOVA) results for Y_1 (Li)

Source	Sum of Squares	df	Mean Square	F-value	p-value	
**Model**	683.51	2	341.75	105.14	< 0.0001	significant
B-Cit	298.05	1	298.05	91.70	< 0.0001	
B^2	379.86	1	379.86	116.87	< 0.0001	
**Residual**	52.01	16	3.25			
Lack of Fit	27.69	12	2.31	0.3795	0.9134	not significant
Pure Error	24.32	4	6.08			
**Cor Total**	735.51	18				

2.3.3 Factor effects on Co leaching

Table S6. The analysis of variance (ANOVA) results for Y_2 (Co)

Source	Sum of Squares	df	Mean Square	F-value	p-value	
**Model**	1128.58	6	188.10	17.29	< 0.0001	significant
A-H_2O_2	0.8213	1	0.8213	0.0755	0.7882	
B-Cit	580.31	1	580.31	53.35	< 0.0001	
C-Na_2EDTA	0.5919	1	0.5919	0.0544	0.8195	
AB	205.49	1	205.49	18.89	0.0010	
BC	164.13	1	164.13	15.09	0.0022	
B^2	199.10	1	199.10	18.30	0.0011	
**Residual**	130.54	12	10.88			
Lack of Fit	60.15	8	7.52	0.4273	0.8577	not significant
Pure Error	70.39	4	17.60			
**Cor Total**	1259.12	18				

2.3.4 Factor effects on Ni leaching

Table S7. The analysis of variance (ANOVA) results for Y3 (Ni)

Source	Sum of Squares	df	Mean Square	F-value	p-value	
**Model**	1970.64	6	328.44	12.80	0.0001	significant
A-H_2O_2	71.76	1	71.76	2.80	0.1202	
B-Cit	974.93	1	974.93	38.01	< 0.0001	
C-Na_2EDTA	277.77	1	277.77	10.83	0.0065	
AC	155.02	1	155.02	6.04	0.0301	
BC	365.87	1	365.87	14.26	0.0026	
B^2	165.49	1	165.49	6.45	0.0259	
**Residual**	307.80	12	25.65			
Lack of Fit	182.13	8	22.77	0.7246	0.6772	not significant
Pure Error	125.67	4	31.42			
**Cor Total**	2278.44	18				

2.3.5 Factor effects on Mn leaching

Table S8. The analysis of variance (ANOVA) results for Y4 (Mn)

Source	Sum of Squares	df	Mean Square	F-value	p-value	
**Model**	2268.15	3	756.05	20.37	< 0.0001	significant
B-Cit	1155.16	1	1155.16	31.13	< 0.0001	
C-Na_2EDTA	497.90	1	497.90	13.42	0.0023	
BC	709.71	1	709.71	19.13	0.0005	
**Residual**	556.62	15	37.11			
Lack of Fit	466.08	11	42.37	1.87	0.2864	not significant
Pure Error	90.54	4	22.64			
**Cor Total**	2824.78	18				

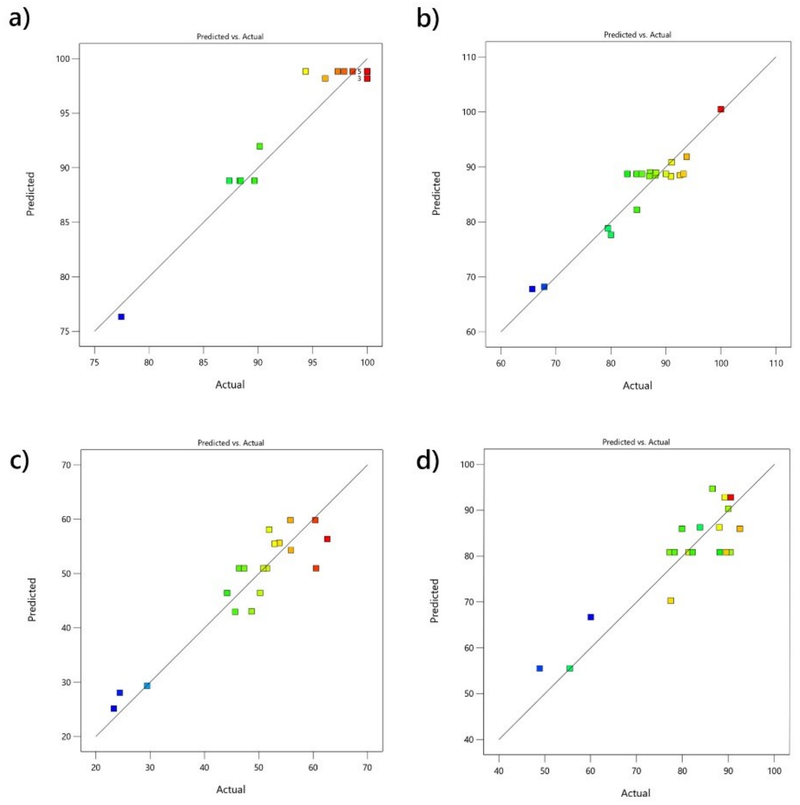

Figure S3. Predicted vs. Actual for the fitted models: (a) Li; (b) Co; (c) Ni; (d) Mn.

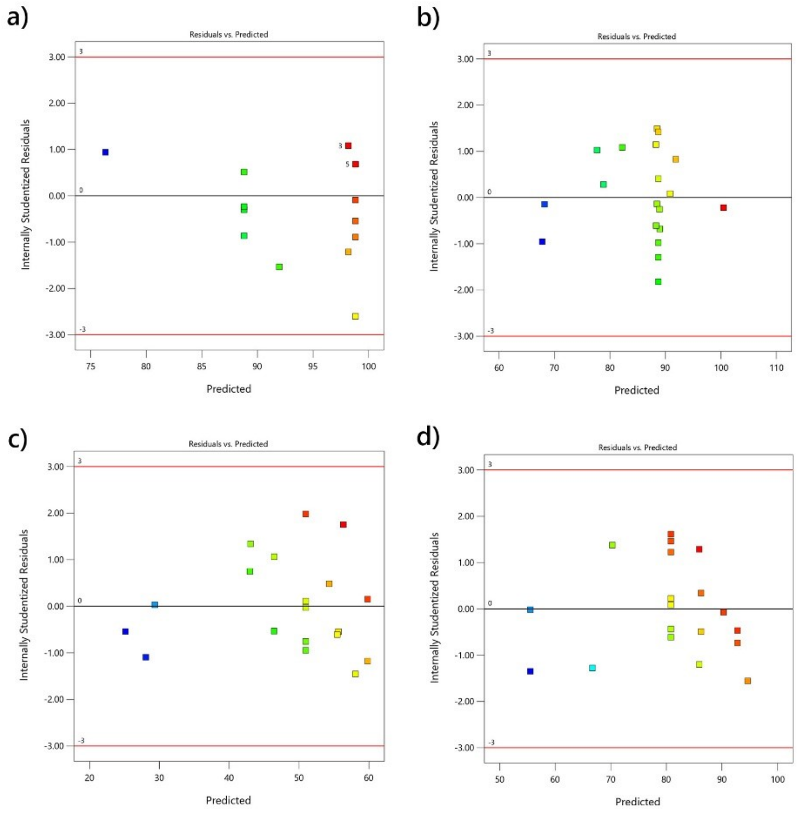

Figure S4. Internally Studentized Residuals vs. Predicted: (a) Li; (b) Co; (c) Ni; (d) Mn.

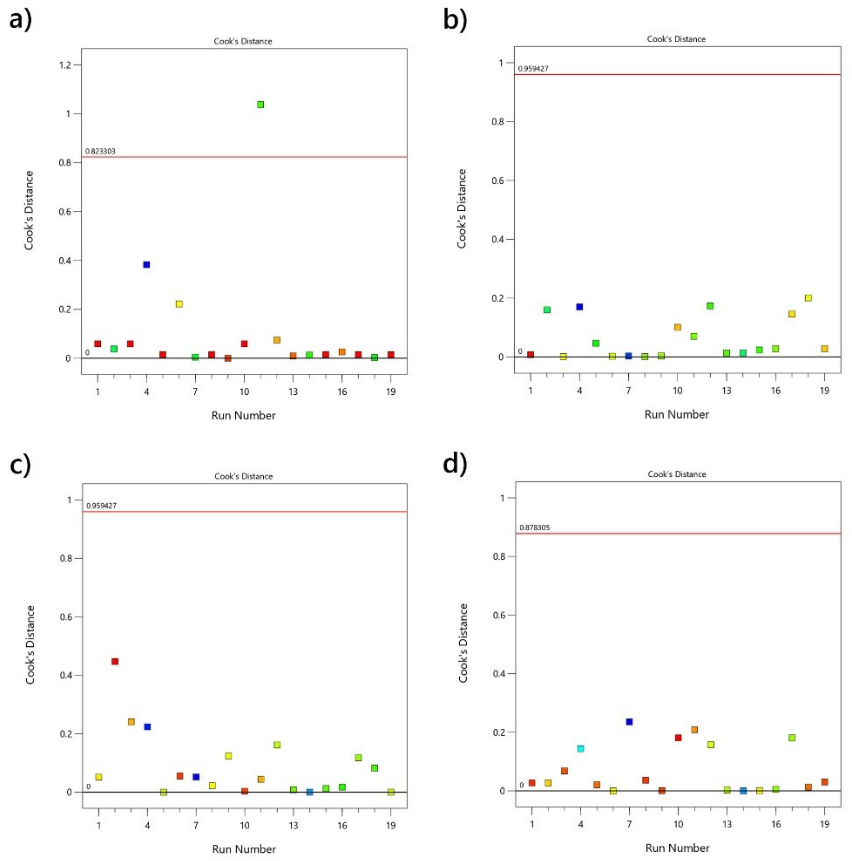

Figure S5. Cook's Distance vs. Run Number (influence diagnostics): (a) Li; (b) Co; (c) Ni; (d) Mn.

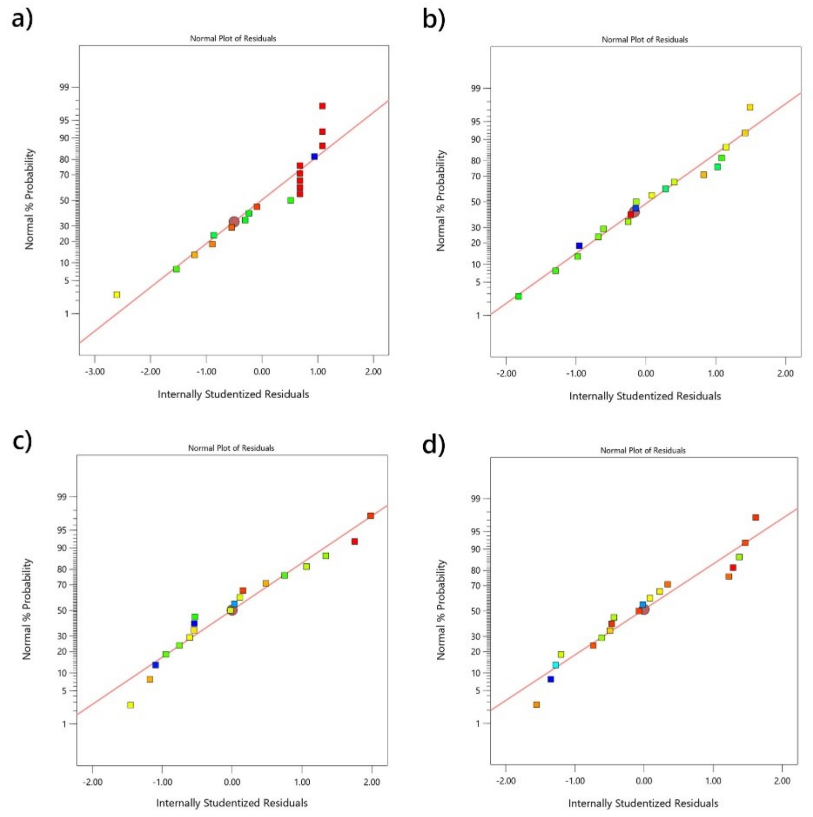

Figure S6. Normal Probability Plot of Internally Studentized Residuals: (a) Li; (b) Co; (c) Ni; (d) Mn.

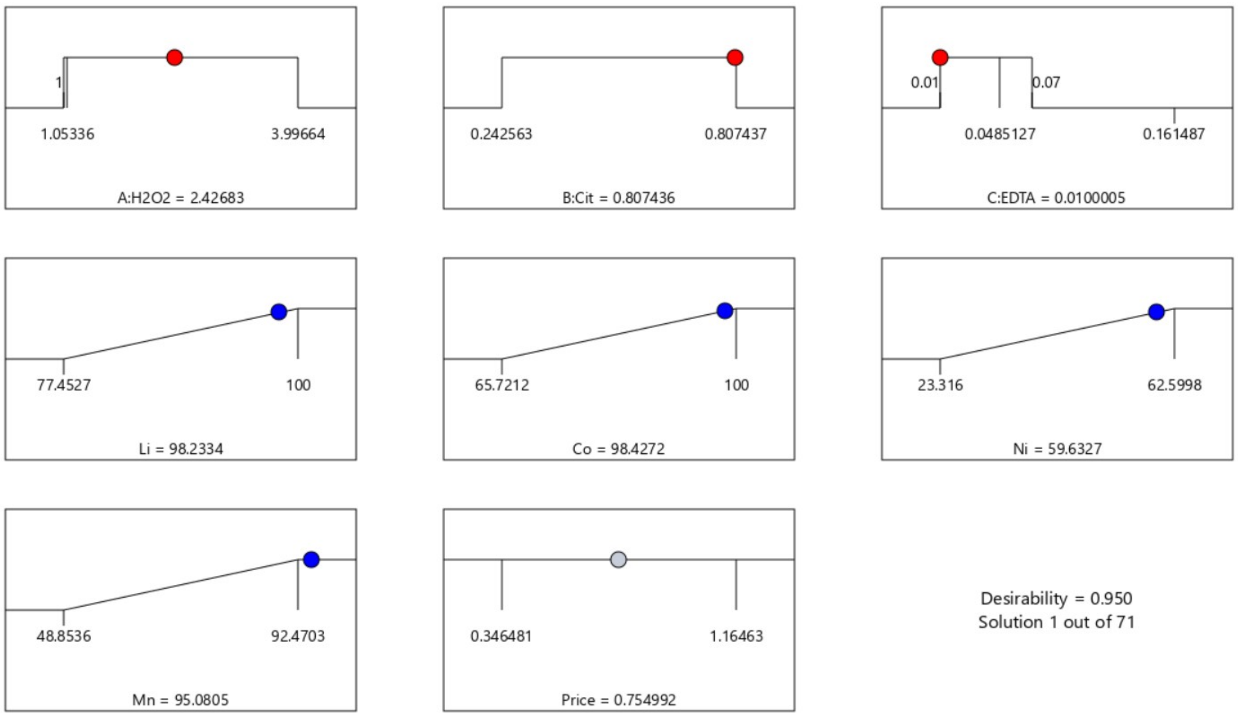

Figure S7. Desirability Ramp Plot (Derringer–Suich) for Multi-Response Optimization — Solution 1/71 (D = 0.950)

3 OPTIMIZATION

Table S9. Mechanistic control map for Li, Co, Ni and Mn in the citrate–EDTA–H_2O_2 system (pH ≈ 5, 50 °C)

Metal	Dominant pathway – Citrate Concentration	H_2O_2 role	Na_2EDTA	Key interactions	Limiting stage/sensibility
**Li**	Acid-complex delithiation, completely controlled by B; pronounced internal optimum for B^2.	Not limiting; medium maintenance; A and its interactions are statistically insignificant	Not limiting at pH>5; secondary chelation.	Not significant interactions (AB, AC, BC — n.s.)	Limit: proton attack on the lattice and mass transfer; sensitivity to the growth of ionic strength/viscosity at high B. Range of cit. conc. ≈ 0.45–0.80 M (R(Li) ≥ 95 %).
**Co**	Citrate-controlled dissolution with an internal maximum (B, B^2 are significant).	Strong synergy with citrate; effective at sufficient B; technologically 1.20–5.00 v/v %	Useful in moderate doses; at high BC, competition is observed.	AB (+) synergy; BC (–) competition; B^2 → optimum	Limit: balance of "oxidative/reductive" dissolution and ligand competition; viscosity/mass transfer at high B and/or C. Range of cit. conc. ≈ 0.60–1.00 M, H_2O_2 ≈ 1.20–5.00 vol.%, [Na_2EDTA] ≈ 0.01M (R(Co) ≥ 95 %).
**Ni**	Joint control by B (with an optimum for B^2) and C.	Linearly insignificant by itself; critical through AC synergy in the presence of Na_2EDTA (recommended 0.05–1.50 vol.%).	Significant positive contribution; rapid chelation of Ni^{2+} suppresses passivation.	AC (+) «redox-handoff»; BC (–) ligand competition; B^2 → optimum.	Limit: risk of Ni(OH)2 passivation with a chelation deficit; avoid high B addnd C zones (due to BC–). Value of cit. conc. ≈ 1.00 M, [Na_2EDTA] ≈ 0.01 – 0.08 M, H_2O_2 ≈ 0.05 – 1.50 vol.%.

**Mn**	Ligand-controlled recovery; linear B and C dominate.	Not limiting in the studied range; secondary role.	Positive contribution; capable of reducing Mn(IV) oxides to soluble Mn(II/III) complexes.	BC (–) – key competition at high joint concentrations.	Limit: rate of complexation and mass transfer; sensitivity to simultaneous growth of B and C (due to BC–). Range of cit. conc. ≈ 0.80–1.00 M, [Na_2EDTA] 0.01 – 0.06 M; H_2O_2 – secondary parameter.

3.1 Establishing the mechanism of metal leaching

Table S10. Kinetic models for lithium leaching

**Temperature**	**Model**	**R^2**	**k**	**n**	**k_1**	**k_2**
30 °C	First-order	0,9832	1,633			
30 °C	Avrami–Erofeev	0,9963	4,776	0,2968		
30 °C	Peleg	0,9942			0,2607	1,005
50 °C	First-order	0,9821	2,218			
50 °C	Avrami–Erofeev	0,9903	10	0,2654		
50 °C	Peleg	0,9922			0,1244	1,014
60 °C	First-order	0,9767	1,777			
60 °C	Avrami–Erofeev	0,9952	9,442	0,2381		
60 °C	Peleg	0,9907			0,2277	1,008

Table S11. Kinetic models for cobalt leaching

**Temperature**	**Model**	**R^2**	**k**	**n**	**k_1**	**k_2**
30 °C	First-order	0,9736	0,0457			
30 °C	Avrami–Erofeev	0,9763	0,0458	0,8832		
30 °C	Peleg	0,9554			12,73	0,964
50 °C	First-order	0,8437	0,08184			
50 °C	Avrami–Erofeev	0,8805	0,09082	0,691		
50 °C	Peleg	0,8502			6,373	0,9614
60 °C	Avrami–Erofeev	0,9565	0,07695	0,6676		
60 °C	Peleg	0,929			7,189	0,9746
60 °C	First-order	0,9139	0,06848			

Table S12. Kinetic models for nickel leaching

**Temperature**	**Model**	**R^2**	**k**	**n**	**k_1**	**k_2**	**α**	**β**
30 °C	Peleg	0,9816			102,4	1,016		
30 °C	Avrami–Erofeev	0,9735	0,005094	0,6896				
30 °C	Elovich	0,9569					0,01657	4,543
50 °C	Peleg	0,984			27,78	1,071		
50 °C	Avrami–Erofeev	0,9649	0,01466	0,5134				
50 °C	Elovich	0,9514					0,129	6,655
60 °C	Peleg	0,9537			24,3	1,191		
60 °C	Elovich	0,8468					0,1738	7,859
60 °C	Avrami–Erofeev	0,8461	0,01093	0,4037				

Table S13. Kinetic models for manganese leaching

Temperature	Model	R^2	k	n	k_1	k_2	α	β
30 °C	Peleg	0,987			55,99	1,158		
30 °C	Avrami–Erofeev	0,9586	0,00585	0,4944				
30 °C	Elovich	0,9525					0,04392	6,246
50 °C	Peleg	0,9847			12,42	1,201		
50 °C	Avrami–Erofeev	0,8984	0,01943	0,3146				
50 °C	Elovich	0,8903					0,9238	9,881
60 °C	Peleg	0,9429			31,18	1,132		
60 °C	Avrami–Erofeev	0,8536	0,01141	0,5145				
60 °C	Elovich	0,8384					0,09878	6,81

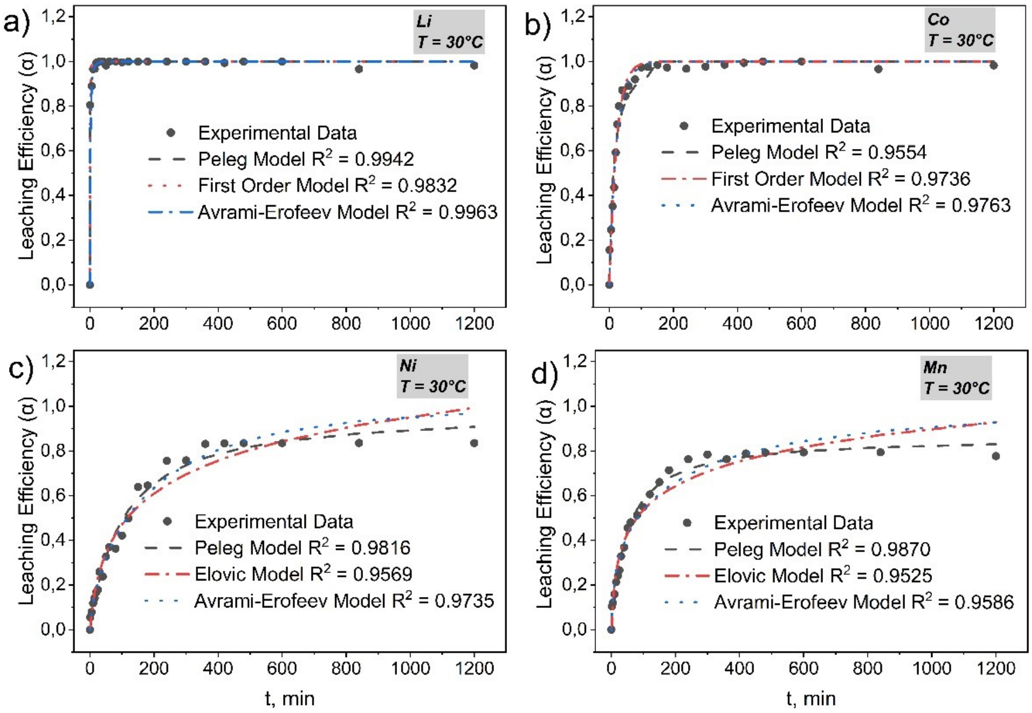

Figure S8. Kinetic curves of Li, Co, Ni and Mn at T = 30°C. Conditions: C(citrates) = 0.8 mol L^{-1}; $C(H_2O_2)$ = 1.2 v/v %; $C(Na_2EDTA)$ = 0.05 mol·L^{-1}; v = 400 rpm; S : L = 1 : 50 (Points – experimental data; dashes – model curves)

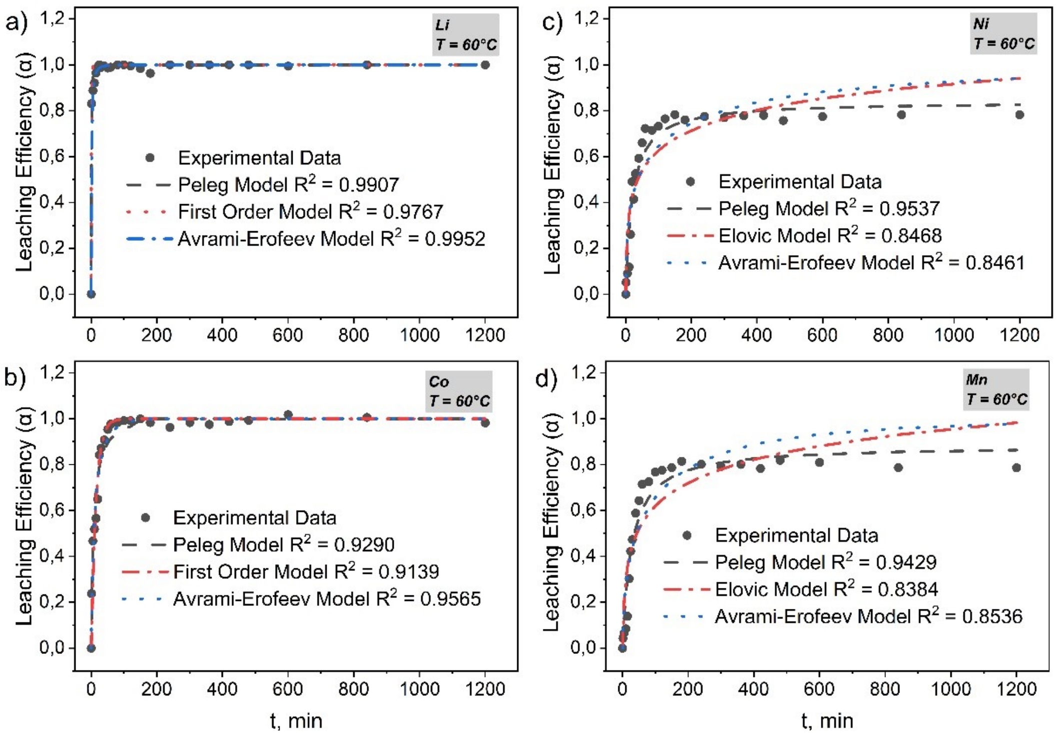

Figure S9. Kinetic curves of Li, Co, Ni and Mn at T = 60°C. Conditions: C(citrates) = 0.8 mol L^{-1}; C(H_2O_2) = 1.2 v/v %; C(Na_2EDTA) = 0.05 mol·L^{-1}; v = 400 rpm; S : L = 1 : 50 (Points – experimental data; dashes – model curves)

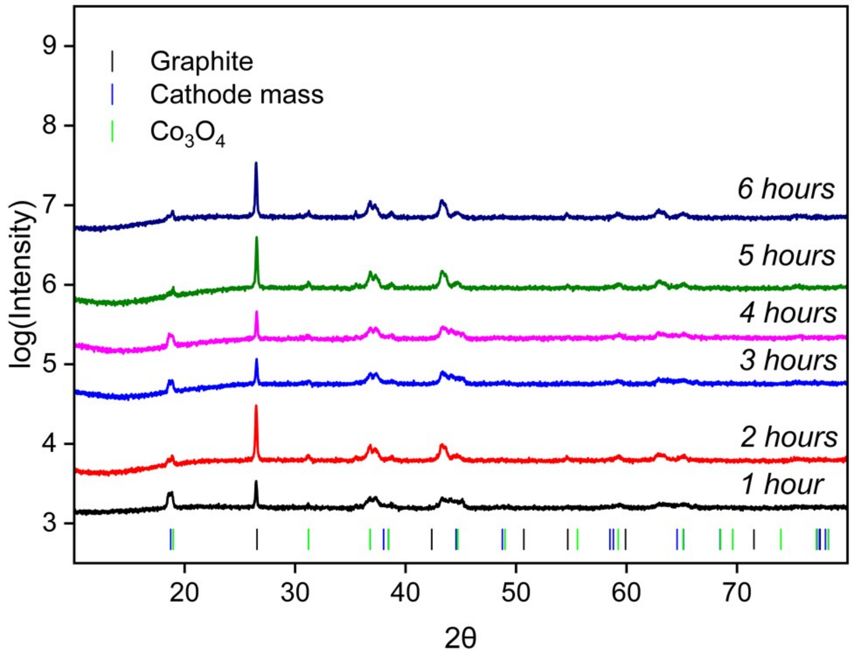

Figure S10. X-ray diffractograms of electrode mass during leaching time in the system of 0.8 mol L^{-1} citrate buffer, 1.2 v/v % H_2O_2 and 0.05 mol L^{-1} Na_2EDTA

3.1.1 Main Chemical Reactions During the Leaching Process

Based on the actual phase composition of the electrode mass after thermal treatment (600°C, 15 h), as confirmed by our XRD and Rietveld analysis, the principal solid phases consist of $LiCoO_2$, LiCoxNiyMnzO_2, metallic nickel, NiO, and graphite.

The leaching process in citrate–EDTA medium at pH ~5 proceeds via the following key reactions:

$LiCoO_2$ leaching:

$$LiCoO_2(s) + H^+(aq) + citrate/EDTA \rightarrow Li^+(aq) + [Co^{2+}(cit/EDTA)]^{n-}(aq) + H_2O \quad (6)$$

Upon leaching process, cobalt is reduced from +3 to +2 with H_2O_2

$LiCo_xNi_yMn_zO_2$ leaching:

$$LiCo_xNi_yMn_zO_2(s) + H^+(aq) + citrate/EDTA \rightarrow Li^+(aq) + [Co^{2+}(cit/EDTA)]^{n-}(aq) + [Ni^{2+}(cit/EDTA)]^{n-}(aq) + [Mn^{2+}(cit/EDTA)]^{n-}(aq) + H_2O \quad (7)$$

Each transition metal ion is reduced to divalent state (+2) and stabilized by the respective ligand.

Metallic nickel reduction and leaching:

$$Ni(s) + H_2O_2(aq) + 2H^+(aq) \rightarrow Ni^{2+}(aq) + 2H_2O \quad (8)$$

$$Ni^{2+}(aq) + citrate/EDTA \rightarrow [Ni^{2+}(cit/EDTA)]^{n-}(aq)$$

Nickel oxide dissolution:

$$NiO(s) + 2H^+(aq) + citrate/EDTA \rightarrow [Ni^{2+}(cit/EDTA)]^{n-}(aq) + H_2O \quad (9)$$

In all stages, charge neutrality is maintained by the concurrent release of Li^+ ions, proton insertion into residual solid phases, and continuous electron redistribution among the transition metal centers during reduction. The resulting solvated complexes remain stably dispersed in solution, preventing reprecipitation.

4. THERMODYNAMICS OF METAL LEACHING PROCESSES FROM SPENT LITHIUM-ION BATTERIES

Thermodynamic investigation of leaching processes is based on a two-level approach, including analysis of equilibrium state functions (ΔH, ΔS, ΔG) and transition state barrier parameters ($\Delta H^‡$, $\Delta S^‡$, $\Delta G^‡$). Equilibrium parameters characterize the overall thermodynamic possibility of metal dissolution and complexation reactions, determining the sign and magnitude of the process driving force. Barrier parameters describe energetic characteristics of the rate-limiting stage, allowing separation of enthalpic and entropic contributions to the activation barrier and, consequently, establishing the nature of kinetic limitations.

Lithium

Lithium demonstrates unique thermodynamic characteristics, distinguished by the minimal enthalpic barrier among the studied metals. At 303.15 K, the activation enthalpy is only 1.39 $kJ \cdot mol^{-1}$, however the process is characterized by extremely negative activation entropy ($\Delta S^‡ = -270.0\ J \cdot mol^{-1} \cdot K^{-1}$)), leading to formation of a significant Gibbs barrier of 83.25 $kJ \cdot mol^{-1}$. Notably, the activation energy increases with temperature rise (88.65 $kJ \cdot mol^{-1}$at 323 K, 91.35 $kJ \cdot mol^{-1}$at 333 K), indicating the dominant contribution of the entropic factor $-T\Delta S^‡$ to process kinetics.

Equilibrium parameters characterize the process as moderately exothermic ($\Delta H = -17.874$ $kJ \cdot mol^{-1}$) with small entropic losses ($\Delta S = -16.078\ J \cdot mol^{-1} \cdot K^{-1}$). Consequently, Gibbs energy remains consistently negative throughout the entire temperature range (−13.355 at 303 K, −11.469 at 323 K, −13.374 $kJ \cdot mol^{-1}$ at 333 K), ensuring thermodynamic feasibility of leaching. Activation energy according to the Avrami model is 20.884 $kJ \cdot mol^{-1}$, which is consistent with autocatalytic

mechanisms characteristic of this model, describing nucleation and growth of reaction centers on particle surfaces.

Table S14. Thermodynamic functions for lithium leaching process in the developed reagent regime

T, K	E_a (Avrami kA), kJ·mol^{-1}	ΔH‡ (Eyring, First-order k), kJ·mol^{-1}	ΔS‡ (Eyring), J·mol^{-1}·K^{-1}	ΔG‡ (Eyring), kJ·mol^{-1}	ΔH (leaching, van't Hoff), kJ·mol^{-1}	ΔS (leaching, van't Hoff), J·mol^{-1}·K^{-1}	ΔG (leaching), kJ·mol^{-1}
303.15	20.884	1.39	−270.0	83.25	−17.874	−16.078	−13.355
323.15				88.65			−11.469
333.15				91.35			−13.374

Cobalt

Cobalt exhibits the most complex thermodynamic picture among the studied metals. Activation enthalpy is 11.37 kJ·mol^{-1} with extremely unfavorable activation entropy (ΔS‡ = −267.5 J·mol^{-1}·K^{-1}), forming the maximum Gibbs barrier of 92.1 kJ·mol^{-1} at 303.15 K. Characteristically, the barrier continues to increase with temperature (97.4 kJ·mol^{-1} at 323 K, 100.0 kJ·mol^{-1} at 333 K), emphasizing the entropy-controlled nature of the rate-limiting stage.

Equilibrium thermodynamics of cobalt demonstrates fundamentally different behavior. Reaction enthalpy is practically zero (ΔH ≈ 0 kJ·mol^{-1}), while the entropic contribution is positive and significant (ΔS = +57.43 J·mol^{-1}·K^{-1}). Consequently, thermodynamic driving force strengthens with temperature increase: from −17.4 kJ·mol^{-1} at 303 K to −19.1 kJ·mol^{-1} at 333 K. Activation energy according to the Avrami model constitutes the minimum value among all metals (16.71 kJ·mol^{-1}), which correlates with preferential description of cobalt kinetics by Avrami-Erofeev and first-order models, indicating a combination of autocatalytic and diffusion mechanisms.

Table S15. Thermodynamic functions for cobalt leaching process in the developed reagent regime

T, K	E_a (Avrami kA), kJ·mol^{-1}	ΔH‡ (Eyring, First-order k), kJ·mol^{-1}	ΔS‡ (Eyring), J·mol^{-1}·K^{-1}	ΔG‡ (Eyring), kJ·mol^{-1}	ΔH (leaching, van't Hoff), kJ·mol^{-1}	ΔS (leaching, van't Hoff), J·mol^{-1}·K^{-1}	ΔG (leaching), kJ·mol^{-1}
303.15	16.71	11.37	−267.5	92.1	0	57.43	−17.4
323.15				97.4			−18.0
333.15				100.0			−19.1

Nickel

Nickel is characterized by intermediate values of barrier parameters: ΔH‡ = 11.19 kJ·mol^{-1}and ΔS‡ = −251.7 J·mol^{-1}·K^{-1} at 303.15 K, forming a Gibbs barrier of 87.4 kJ·mol^{-1}. Similar to other metals, activation energy increases with temperature due to entropic contribution, reaching 96.3 kJ·mol^{-1} at 333 K. These parameters are consistent with preferential description of nickel kinetics by the Peleg model, characterizing processes with saturation and accounting for mass transfer limitations.

Equilibrium thermodynamics of nickel differs by the most pronounced exothermic character (ΔH = −67.98 kJ·mol^{-1}) among all studied metals. However, the process is accompanied by significant entropic losses (ΔS = −189.5 J·mol^{-1}·K^{-1}), leading to decreased thermodynamic driving force upon heating. Gibbs energy decreases in magnitude from −10.42 kJ·mol^{-1} at 303 K to −4.59 kJ·mol^{-1} at 333 K, indicating thermodynamic preference for moderate temperatures. Activation energy according to the Avrami model (17.75 kJ·mol^{-1}) reflects moderate temperature sensitivity of the reaction center nucleation stage.

Table S16. Thermodynamic functions for nickel leaching process in the developed reagent regime

T, K	E_a (Avrami kA), kJ·mol^{-1}	ΔH‡ (Eyring, First-order k), kJ·mol^{-1}	ΔS‡ (Eyring), J·mol^{-1}·K^{-1}	ΔG‡ (Eyring), kJ·mol^{-1}	ΔH (leaching, van't Hoff), kJ·mol^{-1}	ΔS (leaching, van't Hoff), J·mol^{-1}·K^{-1}	ΔG (leaching), kJ·mol^{-1}
303.15	17.75	11.19	−251.7	87.4	−67.98	−189.5	−10.42
323.15				93.4			−7.11
333.15				96.3			−4.59

Manganese

Manganese demonstrates activation enthalpy of 12.82 kJ·mol^{-1} and activation entropy of −245.1 J·mol^{-1}·K^{-1}, forming a Gibbs barrier of 87.2 kJ·mol^{-1} at 303.15 K. Temperature dependence of the barrier follows the general pattern of entropic control, increasing to 95.4 kJ·mol^{-1} at 333 K. Notably, manganese is characterized by maximum activation energy according to the Avrami model (29.64 kJ·mol^{-1}), which is consistent with preferential description of its kinetics by the Peleg model and indicates the most pronounced temperature sensitivity of nucleation and growth stages of reaction centers.

Equilibrium parameters of manganese are characterized by significant exothermicity (ΔH = −50.98 kJ·mol^{-1}) and negative entropic contribution (ΔS = −127.2 J·mol^{-1}·K^{-1}). Similar to nickel, thermodynamic driving force weakens with temperature increase, demonstrating a minimum at 323 K (−6.28 kJ·mol^{-1}) with slight recovery at 333 K (−6.97 kJ·mol^{-1}). Such behavior indicates complex temperature dependence of equilibrium, related to competition between enthalpic and entropic factors.

Table S17. Thermodynamic functions for manganese leaching process in the developed reagent regime

T, K	E_a (Avrami kA), kJ·mol^{-1}	ΔH‡ (Eyring, First-order k), kJ·mol^{-1}	ΔS‡ (Eyring), J·mol^{-1}·K^{-1}	ΔG‡ (Eyring), kJ·mol^{-1}	ΔH (leaching, van't Hoff), kJ·mol^{-1}	ΔS (leaching, van't Hoff), J·mol^{-1}·K^{-1}	ΔG (leaching), kJ·mol^{-1}
303.15	29.64	12.82	−245.1	87.2	−50.98	−127.2	−7.94
323.15				92.8			−6.28
333.15				95.4			−6.97

Thermodynamic analysis demonstrates that metal leaching processes from lithium-ion batteries are controlled by entropic factors at the kinetic level with different equilibrium driving forces. Optimization of technological parameters should account for individual thermodynamic characteristics of each metal, ensuring balance between kinetic and equilibrium factors to achieve maximum leaching efficiency.
